# The endosomal RIN2/Rab5C machinery prevents VEGFR2 degradation to control gene expression and tip cell identity during angiogenesis

**DOI:** 10.1007/s10456-021-09788-4

**Published:** 2021-05-13

**Authors:** Lanette Kempers, Yuki Wakayama, Ivo van der Bijl, Charita Furumaya, Iris M. De Cuyper, Aldo Jongejan, Marije Kat, Anne-Marieke D. van Stalborch, Antonius L. van Boxtel, Marvin Hubert, Dirk Geerts, Jaap D. van Buul, Dirk de Korte, Wiebke Herzog, Coert Margadant

**Affiliations:** 1 Sanquin Research, Plesmanlaan 125, 1066 CX Amsterdam, The Netherlands; 2 Max Planck Institute for Molecular Biomedicine, Roentgenstrasse 20, 48149 Muenster, Germany; 3 Department of Epidemiology and Data Science /Amsterdam Public Health Research Institute, Amsterdam UMC, University of Amsterdam, Meibergdreef 9, 1105 AZ Amsterdam, The Netherlands; 4 Cancer Biology and Genetics and Department of Medicine, Memorial Sloan Kettering Cancer Center, New York, NY 10065 USA; 5 Department of Medical Biology, Amsterdam UMC, University of Amsterdam, Meibergdreef 9, 1105 AZ Amsterdam, The Netherlands; 6 Sanquin Blood Bank, Plesmanlaan 125, 1066 CX Amsterdam, The Netherlands; 7 University of Muenster, Schlossplatz 2, 48149 Muenster, Germany; 8 Angiogenesis Laboratory, Department of Medical Oncology, Amsterdam University Medical Center, location VUmc, De Boelelaan 1117, 1081 HV Amsterdam, The Netherlands; 9 Present Address: Developmental, Stem Cell and Cancer Biology, Swammerdam Institute for Life Sciences, University of Amsterdam, Science Park 904, 1098 XH Amsterdam, The Netherlands

**Keywords:** Endolysosomal trafficking, Early endosomes, Rab5C, Rab GTPases, Notch signaling, VEGFR2, VEGF signaling, Sprouting angiogenesis, Tip cells

## Abstract

Sprouting angiogenesis is key to many pathophysiological conditions, and is strongly regulated by vascular endothelial growth factor (VEGF) signaling through VEGF receptor 2 (VEGFR2). Here we report that the early endosomal GTPase Rab5C and its activator RIN2 prevent lysosomal routing and degradation of VEGF-bound, internalized VEGFR2 in human endothelial cells. Stabilization of endosomal VEGFR2 levels by RIN2/Rab5C is crucial for VEGF signaling through the ERK and PI3-K pathways, the expression of immediate VEGF target genes, as well as specification of angiogenic ‘tip’ and ‘stalk’ cell phenotypes and cell sprouting. Using overexpression of Rab mutants, knockdown and CRISPR/Cas9-mediated gene editing, and live-cell imaging in zebrafish, we further show that endosomal stabilization of VEGFR2 levels is required for developmental angiogenesis in vivo. In contrast, the premature degradation of internalized VEGFR2 disrupts VEGF signaling, gene expression, and tip cell formation and migration. Thus, an endosomal feedforward mechanism maintains receptor signaling by preventing lysosomal degradation, which is directly linked to the induction of target genes and cell fate in collectively migrating cells during morphogenesis.

**Supplementary Information:**

The online version of this article contains supplementary material available (10.1007/s10456-021-09788-4).

## Introduction

Sprouting angiogenesis is crucial for a range of pathophysiological processes including embryonic development, wound healing, tissue remodeling, cancer, and cardiovascular disease [1]. A key pro-angiogenic event is the interaction of vascular endothelial growth factor A (VEGF-A, further referred to as VEGF) with VEGF receptor 2 (VEGFR2), which promotes endothelial proliferation, survival, and migration through activation of the extracellular-signal regulated kinase (ERK) and phosphatidylinositol 3-kinase (PI3-K) pathways [2]. VEGF/VEGFR2 signaling also induces a pro-angiogenic gene expression program and stimulates the selection of specialized ‘tip cells’, which have a distinct morphology and transcriptional signature, and promote the guidance of nascent sprouts [3–7]. Tip cells are followed by ‘stalk cells’, which promote sprout elongation and lumenization [4–7]. VEGF-dependent specification of tip and stalk cells is fine-tuned by activation of Notch signaling, which downregulates VEGFR2 expression to prevent uncontrolled sprouting [7–10], as well as by the decoy receptor VEGFR1, which sequesters VEGF away from VEGFR2 [11–13].

Upon VEGFR2 activation by ligand binding, VEGFR2 is rapidly internalized and subsequently recruited to early endosomes (EEs) [2, 14–18], which are marked by the presence of GTPases of the Rab5 subfamily [19]. Rab GTPases are key regulators of vesicle fusion and protein sorting that are activated by GDP/GTP exchange, catalyzed by guanine nucleotide exchange factors (GEFs) [19–21]. Activation is crucial for Rab localization to intracellular membranes, and for the recruitment of effector proteins [19–21]. From EEs, VEGFR2 is trafficked to lysosomes and degraded, thus accelerating its turnover and terminating VEGF signaling, in a manner dependent on epsin binding to ubiquitinated cytoplasmic motifs in VEGFR2 [16, 22–25]. Alternatively, VEGFR2 is recycled back to the cell-surface in recycling compartments, containing either Rab4 or Rab11 [14, 16, 17].

Internalization of activated VEGFR2 and its subsequent flux through the endosomal system are regulated by endocytic adapters, as well as by cell-surface receptors that associate with VEGFR2 [14, 23, 25–31]. Although it is known that VEGFR2 can signal from endosomal compartments [2, 18], the interrelation between VEGFR2 endocytosis and signaling is complex. For example, ephrin B2 knockout impairs both VEGFR2 endocytosis and signaling [30, 31], whereas the targeted deletion of epsins or dynamin blocks VEGFR2 internalization but not signaling [28, 32]. Furthermore, it is important that VEGFR2-containing endosomes progress normally through the endosomal system, as delayed trafficking exposes internalized VEGFR2 to selective dephosphorylation by tyrosine phosphatase PTP1b, leading to reduced activation of the ERK, but not PI3-K pathway, and impaired arteriogenesis [26, 33, 34].

While these findings emphasize the importance of endocytosis for VEGF signaling, less is known about the mechanisms and factors that maintain endosomal VEGFR2 levels and prevent premature degradation. Studies in zebrafish have shown that Rab5C is particularly highly expressed in endothelium and important for endothelial Notch trafficking [35–38], but its role in VEGFR2 traffic is unknown. Moreover, it is unclear if and how endosomal VEGF signaling regulates the induction of VEGF target genes, or the acquisition of tip versus stalk cell properties during sprouting angiogenesis.

Here we show that the regulated maintenance of internalized VEGFR2 and its diversion from the degradation pathway is tightly linked to the induction of VEGF target genes, and is crucial for tip cell specification and endothelial cell migration. The endosomal VEGFR2 pool is protected from degradation by Rab5C, which is recruited to endosomes by the GEF RIN2. Finally, we show by a number of in vitro and in vivo approaches that manipulation of the RIN2/Rab5C machinery leads to premature VEGFR2 degradation, thus disturbing normal endothelial VEGF/Notch signaling and VEGF-dependent gene expression, generation of functional tip cells, and sprouting angiogenesis. In summary, an endosomal feedforward loop controlled by RIN2/Rab5C prevents VEGFR2 degradation, and maintains a VEGF signaling window required for VEGF-induced gene expression, tip/stalk cell specification, and vascular sprouting.

## Results

### Rab5C protects VEGFR2 from VEGF-induced lysosomal degradation

**Fig. 1:**
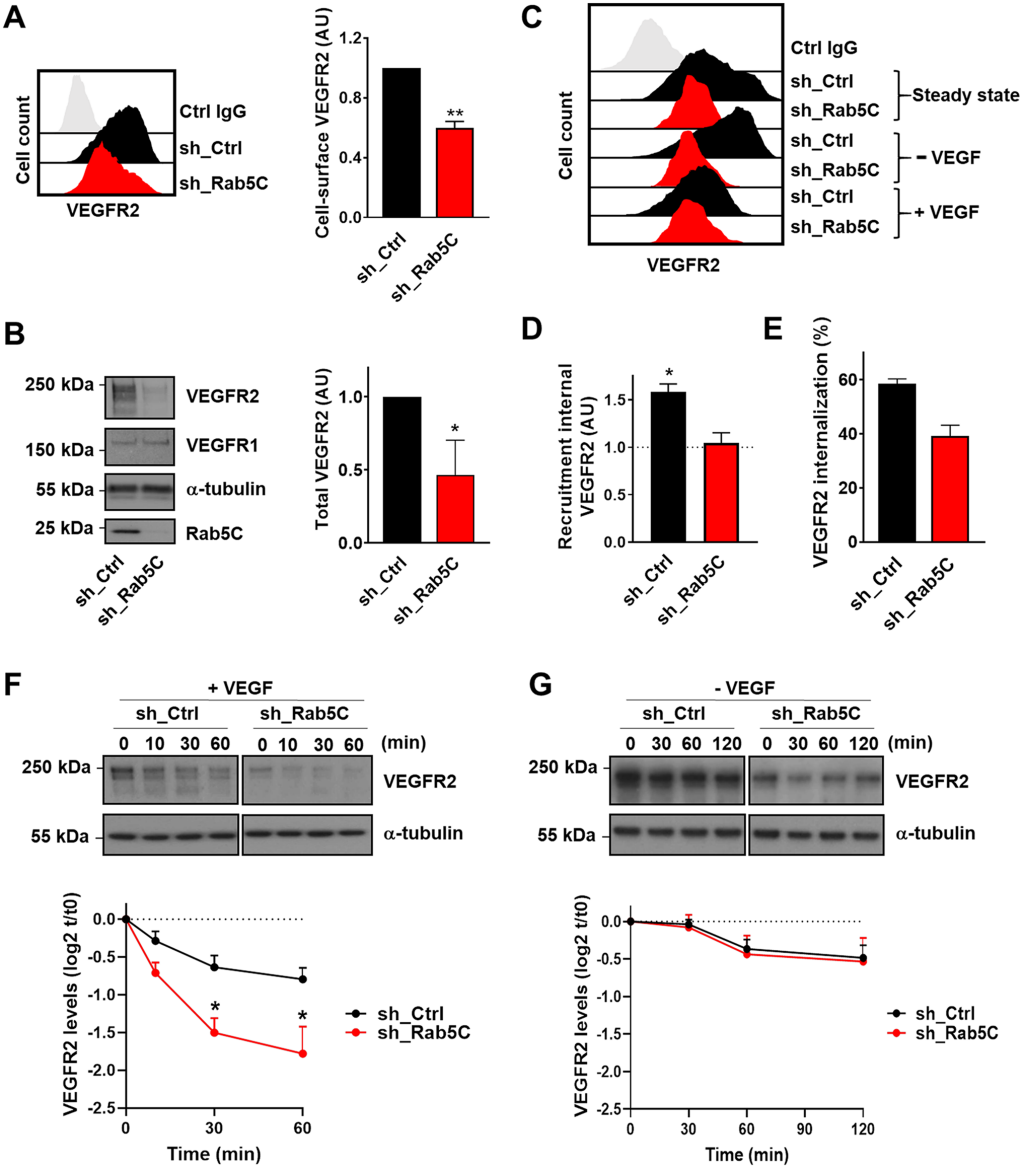
Rab5C protects endosomal VEGFR2 from VEGF-induced lysosomal degradation. **a** Cell-surface levels of VEGFR2 in sh_Ctrl and sh_Rab5C HUVECs analyzed by flow cytometry. (*left*) Histograms of a representative experiment, (*right*) quantification (means + SEM) of 4 independent experiments. Mean fluorescence intensities are expressed relative to those in sh_Ctrl cells. **b** Western blot analysis (*left*) of sh_Ctrl and sh_Rab5C HUVEC lysates probed for VEGFR2 and VEGFR1, with α-tubulin serving as a loading control. Blots are representative of 3 independent experiments. Quantification (*right*) shows the relative VEGFR2 levels normalized to total protein content. Levels in sh_Ctrl cells were set to 1. Results are means + SEM of 3–4 independent experiments. **c** sh_Ctrl and sh_Rab5C HUVECs were starved for 30 min and then either left untreated or stimulated for 30 min with 50 ng/ml VEGF, whereafter the cell-surface levels of VEGFR2 were analyzed by flow cytometry. **d** Recruitment of VEGFR2 upon starvation was calculated as the increase in surface levels with respect to those in steady-state. Results are means + SEM of 3 independent experiments. **e** VEGF-induced VEGFR2 internalization was calculated for sh_Ctrl and sh_Rab5C HUVECs. Mean fluorescence intensities were normalized to steady-state levels. Results are means + SEM of 3–4 independent experiments. **f, g** HUVECs were starved overnight and subsequently stimulated with 50 ng/ml VEGF (**f**) or maintained in starvation medium for the indicated times (**g**). Lysates were subjected to Western blot analysis for VEGFR2, with α-tubulin as a loading control. Blots are representative of 4–5 individual experiments. Quantification of Western blots shows the decline in VEGFR2 levels, expressed relative to the levels at t = 0. Values represent means + SEM of 4–5 independent experiments

We first depleted Rab5C from human umbilical vein endothelial cells (HUVECs) with shRNA pools (leading to ~ 90% reduction at the mRNA level; Fig S1A), and assessed the cell-surface levels of VEGFR2 by flow cytometry. Intriguingly, VEGFR2 surface levels in steady-state HUVECs (growing in the presence of VEGF) were consistently reduced two-fold in sh_Rab5C cells, compared to HUVECs expressing scrambled sequences as a control (sh_Ctrl) (Fig. 1a). The decrease in VEGFR2 was further confirmed by quantification of the total VEGFR2 protein levels by Western blotting (Fig. 1b). In contrast, VEGFR1 protein levels were not affected, suggesting that VEGFR1 is not regulated by Rab5C (Fig. 1b; Fig S1B). We then investigated if and how Rab5C regulates VEGFR2 endocytic recycling using flow cytometry. To test whether the internal pool of VEGFR2 can be recruited normally to the plasma membrane, we starved HUVECs for 30 min, a time-frame that allows mobilization of the internal VEGFR2 pool to the plasma membrane but is too short to induce strong changes in de novo protein synthesis. Starvation significantly increased the cell-surface levels of VEGFR2 compared to those in steady-state in sh_Ctrl but not sh_Rab5C cells, indicating that the recruitment from intracellular compartments was impaired (Fig. 1c, d). VEGF induced VEGFR2 endocytosis in both sh_Ctrl and sh_Rab5C cells, although in the latter the internalization was slightly reduced (Fig. 1c, e). The endosomal pool of VEGFR2 is protected from degradation in the absence of ligand, while VEGF stimulation results in rapid degradation [16, 22]. Therefore, we hypothesized that the reduced levels of VEGFR2 in Rab5C-depleted cells were due to increased degradation. To assess VEGFR2 degradation, HUVECs were deprived of growth factors overnight and then stimulated with VEGF, which resulted in a rapid decrease of up to 40% of VEGFR2 levels in 30 min (Fig. 1f), consistent with previous reports [16, 22]. Strikingly, VEGFR2 degradation was enhanced twofold in Rab5C-depleted cells, confirming our hypothesis that the reduction in VEGFR2 levels in these cells is caused by increased degradation (Fig. 1f). Moreover, inhibition of lysosomal proteases blocked VEGF-induced degradation, and nullified the differences in VEGFR2 degradation between sh_Ctrl and sh_Rab5C cells (Fig S1C). In HUVECs that were maintained in the absence of VEGF, VEGFR2 levels declined only modestly (20% over 120 min), and this was not significantly altered by depletion of Rab5C (Fig. 1g).

In summary, these results show that Rab5C protects the endosomal VEGFR2 pool from VEGF-induced lysosomal degradation.

### Rab5C regulates VEGF signaling, the VEGF-induced immediate transcriptome, and endothelial cell sprouting

**Fig. 2:**
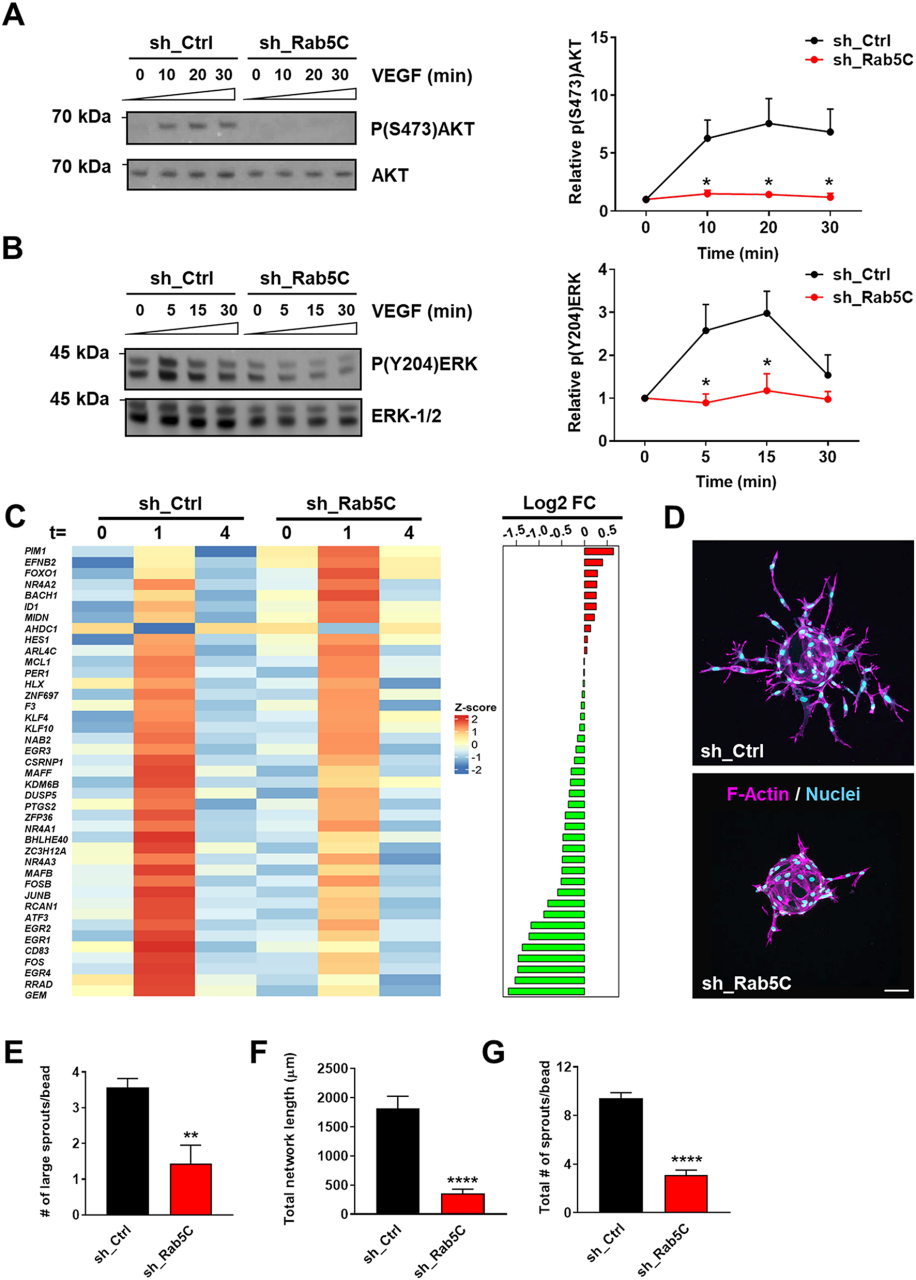
Rab5C regulates endosomal VEGF signaling, the immediate VEGF transcriptome, and sprouting angiogenesis. **a** Western blot analysis (*left*) and quantification (*right*) of phosphorylation of (S473)AKT and total AKT levels at 0, 10, 20, and 30 min of VEGF stimulation (50 ng/ml). **b** Western blot analysis (*left*) and quantification (*right*) of (Y204)ERK-1/2 phosphorylation and total ERK-1/2 levels at 0, 5, 15, and 30 min of VEGF stimulation (50 ng/ml). Representative blots are shown, α-tubulin served as a loading control. Quantifications are means + SEM from 3 independent experiments and expressed relative to t = 0. (**c**) HUVECs were starved overnight (t = 0), then stimulated with VEGF (50 ng/ml) for 1 h (t = 1) or 4 h (t = 4). VEGF-induced gene expression was then assessed by RNA-seq. Genes have been ordered based on the difference in mean log2 FC between sh_Ctrl and sh_Rab5C. Heatmap shows the corresponding centered and scaled mean expression values for VEGF-induced genes at the indicated time-points. Differences in mean log2 fold change (FC) between sh_Ctrl and sh_Rab5C after 1 h of VEGF stimulation are indicated on the right. Genes that have a higher mean expression in sh_Rab5C with respect to sh_Ctrl are indicated in red, those that have a lower mean expression in green. (**d**) Sprouting of sh_Ctrl (*top*) and sh_Rab5C (*bottom*) HUVECs from collagen-coated beads into fibrin gels. Cells were fixed at 48 h, stained for F-actin (*magenta*) and nuclei (*cyan*), and visualized by confocal microscopy. Representative images are shown (maximum projections from z-stacks). Scale bar, 75 μm. **e** Quantification of the average number of large sprouts/bead. Values represent the means + SEM of 3 independent experiments (20 beads per experiment). **f** Quantification of the total network length and **g** the total number of sprouts. A representative experiment is shown. Values represent the means + SEM of *n* = 16 beads per condition

We next investigated if and how Rab5C regulates VEGF signaling. For this purpose, sh_Ctrl and sh_Rab5C HUVECs were starved overnight and then stimulated with VEGF, whereafter AKT phosphorylation was assessed as a read-out for PI3-K-mediated VEGF signaling. VEGF triggered robust and persistent AKT phosphorylation within 10 min in sh_Ctrl cells, which was almost completely abolished in Rab5C-depleted cells, indicating that Rab5C is required for VEGF-induced PI3-K signaling (Fig. 2a). We also investigated VEGF-induced ERK-1/2 phosphorylation. In sh_Ctrl cells, robust induction of ERK-1/2 phosphorylation was observed within 5 min after VEGF addition and persisted for up to 30 min, while this was significantly reduced in sh_Rab5C cells (Fig. 2b).

We then assessed if and how the reduced VEGFR2 signaling observed upon depletion of Rab5C impacts on VEGF-regulated gene expression by mRNA sequencing analysis (RNA-seq). Differential expression was assessed by empirical Bayes analysis followed by correction of *p* values for multiple testing using the Benjamini–Hochberg false discovery rate (FDR), with a cut-off adjusted *p* value of 0.05. Using these criteria, the expression of 40 genes was significantly induced and 1 gene was repressed in sh_Ctrl cells after 1 h (t = 1) of VEGF stimulation, while after 4 h (t = 4) the expression of these genes was largely back to base-line levels (Table S1 and Fig. 2c). These kinetics are comparable with those found in previous reports [39–41]. The induced target genes include well-known immediate VEGF targets, 58% of which encoding transcriptional regulators such as Krüppel-like factors, nuclear receptors of the NR4A family, and members of the Fos/Jun and early growth response protein families, also consistent with earlier findings (Table S1 and Fig. 2c) [39–41]. Strikingly, expression of a large number of VEGF target genes (34 out of 41) was altered in sh_Rab5C cells (Fig. 2c), and geneset enrichment analysis revealed that at t = 1, expression of the ‘VEGF transcriptome’ was significantly different with respect to that in sh_Ctrl cells (*p* = 3.12*10^–4^, FDR = 1.52*10^–2^).

We then assessed the role of Rab5C in sprouting angiogenesis, using the fibrin bead assay [42, 43]. Quantification of the extent of sprouting after 48 h revealed that depletion of Rab5C strongly impaired sprouting, by affecting both the numbers of formed sprouts and their length (Fig. 2d–g). In addition, tip cells clearly formed multiple filopodia in the sh_Ctrl population, a process stimulated by VEGF signaling, whereas sh_Rab5C tip cells were on average more ‘blunted’ (Fig. 2d).

Together, these data show that Rab5C promotes endosomal VEGFR2 signaling and full induction of the immediate VEGF-induced transcriptome, and that Rab5C is required for efficient sprouting angiogenesis.

### Rab5C promotes tip cell formation

**Fig. 3:**
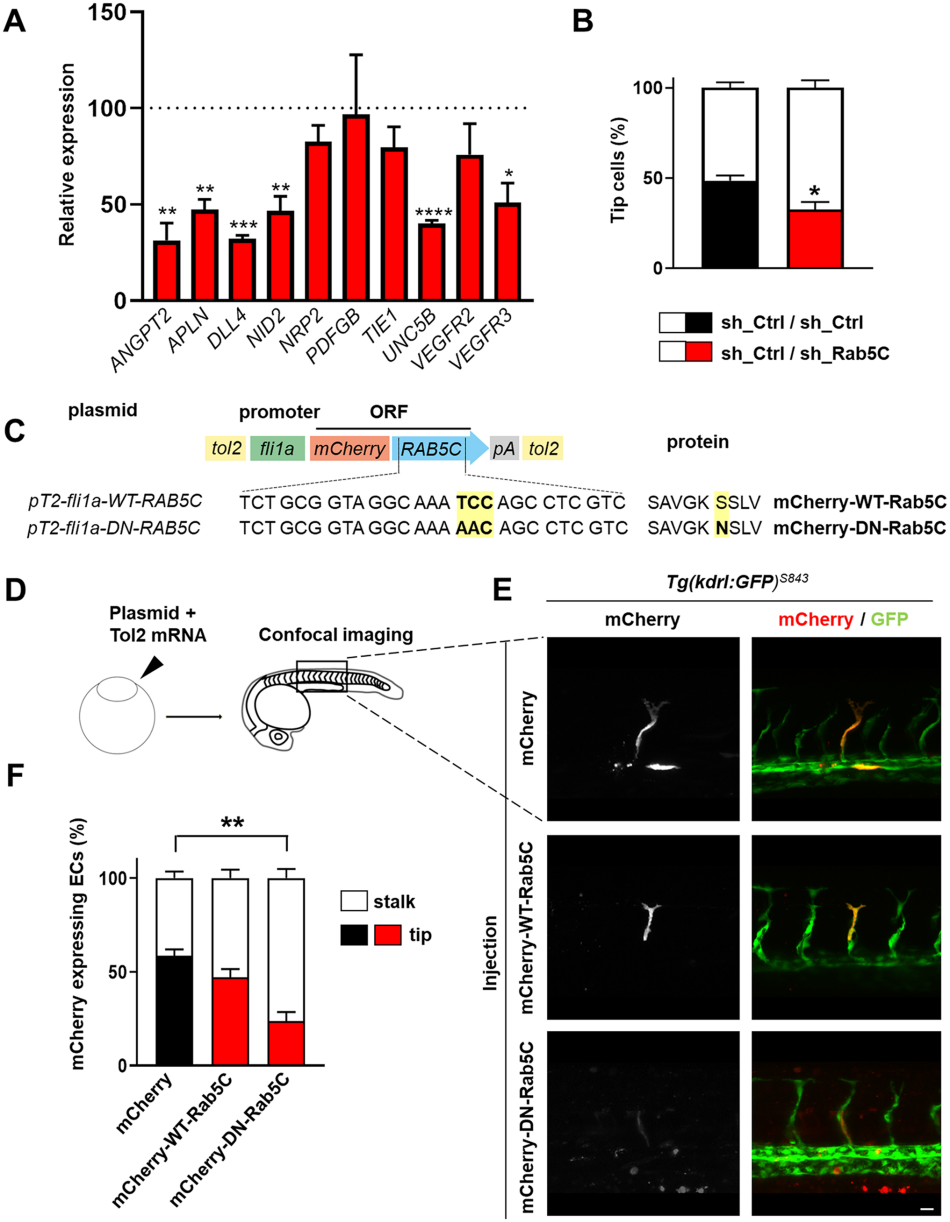
Rab5C is essential for tip cell formation. **a** sh_Ctrl and sh_Rab5C HUVECs were subjected to qPCR analysis for the indicated tip cell markers. Data are means + SEM of 3–5 individual experiments and are expressed relative to the means in sh_Ctrl cells (indicated by dashed line). **b** sh_Ctrl and sh_Rab5C cells were differentially labeled using CellTracker dyes, whereafter they were mixed in a 1:1 ratio, adhered to collagen-coated beads, and subjected to VEGF-stimulated sprouting in fibrin gels. After 48 h, cells were fixed and processed for confocal microscopy. Quantification of the number of tip cells was performed by counting the tip cells from confocal z-stacks and normalizing to the total number of cells in that color. Results shown are the means + SEM of 3 pooled independent experiments. Between 8 and 15 beads (containing on average 14 tip cells/bead) were analyzed per condition per experiment. **c** Schematic of the constructs used for zebrafish experiments. **d** Constructs encoding mCherry-tagged WT or DN Rab5C were injected together with *tol2* transposase mRNA into *Tg(kdrl:GFP)*^*s843*^ zebrafish embryos at the single-cell stage to achieve stable mosaic overexpression in the vasculature. The ISVs were analyzed at 32 hpf. **e** Maximum projections of z-stacks obtained by confocal microscopy of *Tg(kdrl:GFP)*^*s843*^ embryos expressing mCherry, mCherry-WT-Rab5C, or mCherry-DN-Rab5C, showing the position of positive cells in the ISVs at 32 hpf. Scale bar, 20 μm. **f** Quantification of the distribution of mCherry, mCherry-WT-Rab5C, or mCherry-DN-Rab5C positive cells*.* Percentages were calculated per embryo. Shown are the means + SEM. Statistical significance indicates tip cell positioning compared to the mCherry control (mCherry: *N* = 12 embryos, *n* = 123 cells; mCherry-WT-Rab5C: *N* = 17, *n* = 148; mCherry-DN-Rab5C: *N* = 14, *n* = 82)

Because VEGF signaling and the expression of certain VEGF targets is important for the induction and maintenance of tip cells during sprouting angiogenesis, we next addressed whether the reduced sprouting observed in the absence of Rab5C is due to differences in VEGF-induced tip cell specification. For this purpose, we first investigated the expression of a panel of genes commonly associated with tip cell identity, including *ANGPT2*, *APLN*, *DLL4*, *NID2*, *NRP2*, *PDGFB*, *TIE1*, *UNC5B*, *VEGFR2*, and *VEGFR3* [6, 7]. Intriguingly, depletion of Rab5C significantly suppressed the transcription of *ANGPT2*, *APLN*, *DLL4*, *NID2*, *UNC5B*, and *VEGFR3*, while the expression of *NRP2*, *TIE1*, and *VEGFR2* was mildly but not significantly decreased (Fig. 3a). Of note, *PDGFB* expression appeared not to be regulated at all by Rab5C.

We then assessed tip cell formation in mosaic sprouting assays, by differentially labeling sh_Ctrl and sh_Rab5C cells using two distinct CellTracker dyes, mixing them in a 1:1 ratio, and determining the fraction of tip cells formed by each population after 48 h (Fig. 3b). The knockdown of Rab5C significantly reduced the numbers of tip cells in this assay (Fig. 3b). Similar results were obtained using a pool of mixed shRNAs (Fig. 3b), two different individual shRNAs (Fig S2A–C), and when the dyes were swapped between conditions (Fig S2B).

To address the role of Rab5C in an in vivo model system for sprouting angiogenesis, we next analyzed if Rab5C regulates intersegmental vessel (ISV) development in zebrafish. The zebrafish *rab5c* gene is highly homologous to its human counterpart, suggesting an important function in the vasculature [35, 37, 38]. We first generated a dominant-negative (DN) mutant Rab5C protein, carrying a single substitution (S35N) in the GTP-binding pocket (Fig S3A). DN Rab mutants sequester GEFs but fail to bind GTP, and therefore cannot be activated [44]. The mutant was fused to mCherry and expressed in HUVECs to test its ability to localize on endosomes. Wild-type (WT) Rab5C localized predominantly on endosomes, some of which were positive for EEA-1, while occasional distribution to the Golgi network was also observed, using Trans-Golgi Network (TGN)46 as a marker (Fig S3B). In contrast, the mutant localized predominantly at the Golgi and hardly on endosomes (Fig S3B). We then cloned the mCherry-tagged WT or DN human *RAB5C* gene into a construct that integrates via Tol2-mediated transgenesis and is driven by the zebrafish *fli-1a* promoter, to achieve expression specifically in vessels (Fig. 3c) [45]. The construct was injected into single-cell stage *Tg(kdrl:GFP)*^*s843*^ zebrafish embryos expressing GFP in all vascular endothelial cells, and mCherry-positive endothelial cells were examined in developing ISVs at 30–32 h post-fertilization (hpf) (Fig. 3d, e) [46, 47]. As these injections result in mosaic expression of mCherry-Rab5C in endothelial cells, mCherry-positive endothelial cells were scored for their position within the developing vessels (tip, stalk, or dorsal aorta; Fig S4A). The distribution of WT-Rab5C positive cells was similar to that of mCherry-expressing cells (Fig. 3f and Fig S4B). However, cells expressing DN-Rab5C were less commonly found in the tip cell position (22% versus 56% in the mCherry control), and most DN-Rab5C positive cells localized to the stalk or the dorsal aorta (Fig. 3f and Fig S4B).

Altogether, our findings indicate that Rab5C is required for tip cell formation, with Rab5C expressing cells having a competitive advantage for tip cell positioning over cells with impaired Rab5C function.

### Rab5C is required for maintenance of Vegfr2 levels, Vegf/Notch signaling, and sprouting angiogenesis in zebrafish

We showed by mosaic overexpression of DN-Rab5C that Rab5C activation is required for tip cell formation in vivo. While these experiments allowed us to identify the preference for tip or stalk cell position in a competitive situation, we also analyzed the effects of DN-Rab5C overexpressed simultaneously in all endothelial cells, by generating transgenic *Tg(kdrl:GFP)*^*s843*^ embryos with vascular-specific expression of human mCherry-WT-Rab5C (*Tg(fli1a:mCherry-WT-hRAB5C)*^*mu227*^, or mCherry-DN-Rab5C (*Tg(fli1a:mCherry-DN-hRAB5C)*^*mu228*^. Similar to our findings in HUVECs, we observed localization of WT Rab5C protein in rapidly moving vesicles in endothelial cells in vivo (Fig. S4C), while the DN mutant was mostly absent from these vesicles but was instead retained in a perinuclear compartment, most likely the Golgi (Fig. S4C). Indeed, quantification confirmed that the number of mCherry-positive vesicles in DN-Rab5C-expressing ISV cells was strongly decreased, compared to WT-Rab5C expressing cells (Fig. S4D).

**Fig. 4:**
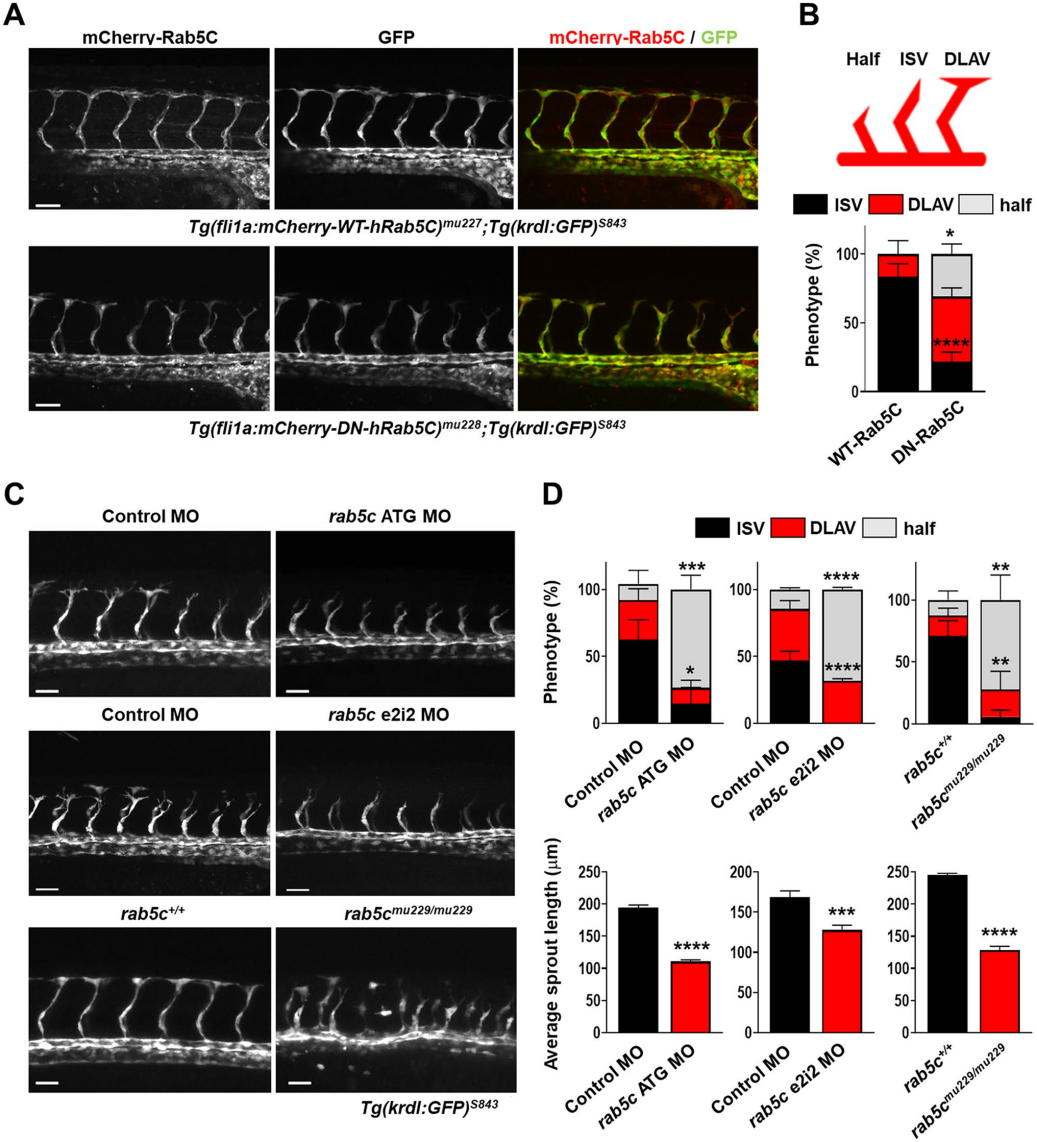
Rab5C is required for sprouting angiogenesis in vivo. **a** Maximum projections of z-stacks obtained by confocal microscopy showing ISV formation in transgenic *Tg(kdrl:GFP)*^*s843*^ zebrafish embryos stably expressing mCherry-WT-Rab5C (*top*) or mCherry-DN-Rab5C (*bottom*). Scale bars, 40 μm. **b** (*top*) ISV formation was scored as indicated, (*bottom*) graph show the results of a representative experiment. Percentages were calculated per embryo (mCherry-WT-Rab5C: *N* = 10 embryos, *n* = 72 ISVs; mCherry-DN-Rab5C: *N* = 12 embryos, *n* = 88 ISVs). Statistical significance indicates DLAV and ‘half’ phenotypes compared to mCherry-WT-Rab5C. **c** Maximum projections of z-stacks obtained by confocal microscopy showing ISV formation in *Tg(kdrl:GFP)*^*s843*^ zebrafish embryos injected with Control or *rab5c* ATG MO (*top*), Control or *rab5c* e2i2 MO (*middle*), or *rab5c*^+*/*+^ versus *rab5c*^*mu229/mu229*^ embryos (*bottom*). Scale bars, 40 μm. **d** ISV development (*top*) as well as average sprout length (*bottom*) were scored for the indicated conditions at 30–32 hpf (6–10 embryos were analyzed per condition, with 6–8 ISVs per embryo). Statistically significant differences in DLAV and ‘half’ phenotypes, compared to Control MO or *rab5c*^+*/*+^, are indicated

We then scored ISV formation at 30–32 hpf, a time-point at which normal ISV endothelial cells have migrated dorsally and interconnected, to form the dorsal longitudinal anastomotic vessel (DLAV) [46, 47]. The survival of DN-Rab5C-expressing embryos was compromised at later stages but not before 30–32 hpf, thus allowing analysis of the developing ISVs. Without WT cells taking over the tip cell function, we observed an impairment of endothelial cell migration in the ISVs of embryos expressing DN-Rab5C (Fig. 4a). While in WT-Rab5C-expressing embryos ISV development was characterized by 83.3% reaching the DLAV and 16.7% migrating nearly to the top, DN-Rab5C-overexpressing endothelial cells migrated only to the level of the horizontal myoseptum (‘half’) in 31% of the embryos, with 47.3% reaching the ISV length, and only 21.7% reaching the DLAV (Fig. 4b).

To disrupt zebrafish Rab5c function in vivo via additional methods, we also used Morpholino (MO)-mediated block of *rab5c* translation (*rab5c* ATG MO) or splicing (*rab5c* e2i2 MO), as well as CRISPR/Cas9-mediated genome modification to induce mutations in the *rab5c* gene (Fig. S5A–E). A heterozygous *rab5c* mutant line was generated by injections of *rab5c* specific guide RNAs (gRNAs) and Cas9 protein, growth to adulthood, identification of germline-transmitting adults, growth of the F1 generation, and identification of the mutation. Detailed genomic analysis revealed that the resulting *rab5c* mutant (designated *rab5c*^*mu229*^) contained a premature stop codon, thus terminating protein synthesis at 29 (instead of 221) amino acids (Fig. S5D,E). We then analyzed vascular development in MO-injected embryos and in the homozygous mutant offspring of *rab5c*^+/*mu229*^ parents. Similar to the DN-Rab5C-expressing embryos, the homozygous mutants died during later stages of development. We observed mispatterning of the ISVs and impaired dorsal migration in individual ISV sprouts in embryos injected with *rab5c* MOs (compared to Control MOs), as well as in *rab5c*^*mu229/mu229*^ embryos (compared to *rab5c*^+*/*+^ siblings) (Fig. 4c,d), thus confirming that Rab5c is required for tip cell formation and normal ISV migration in vivo. Additionally, abnormal sprouting angiogenesis was also revealed by live imaging of ISV development using time-lapse microscopy (Movies S1,S2 and Fig. S6A).

**Fig. 5:**
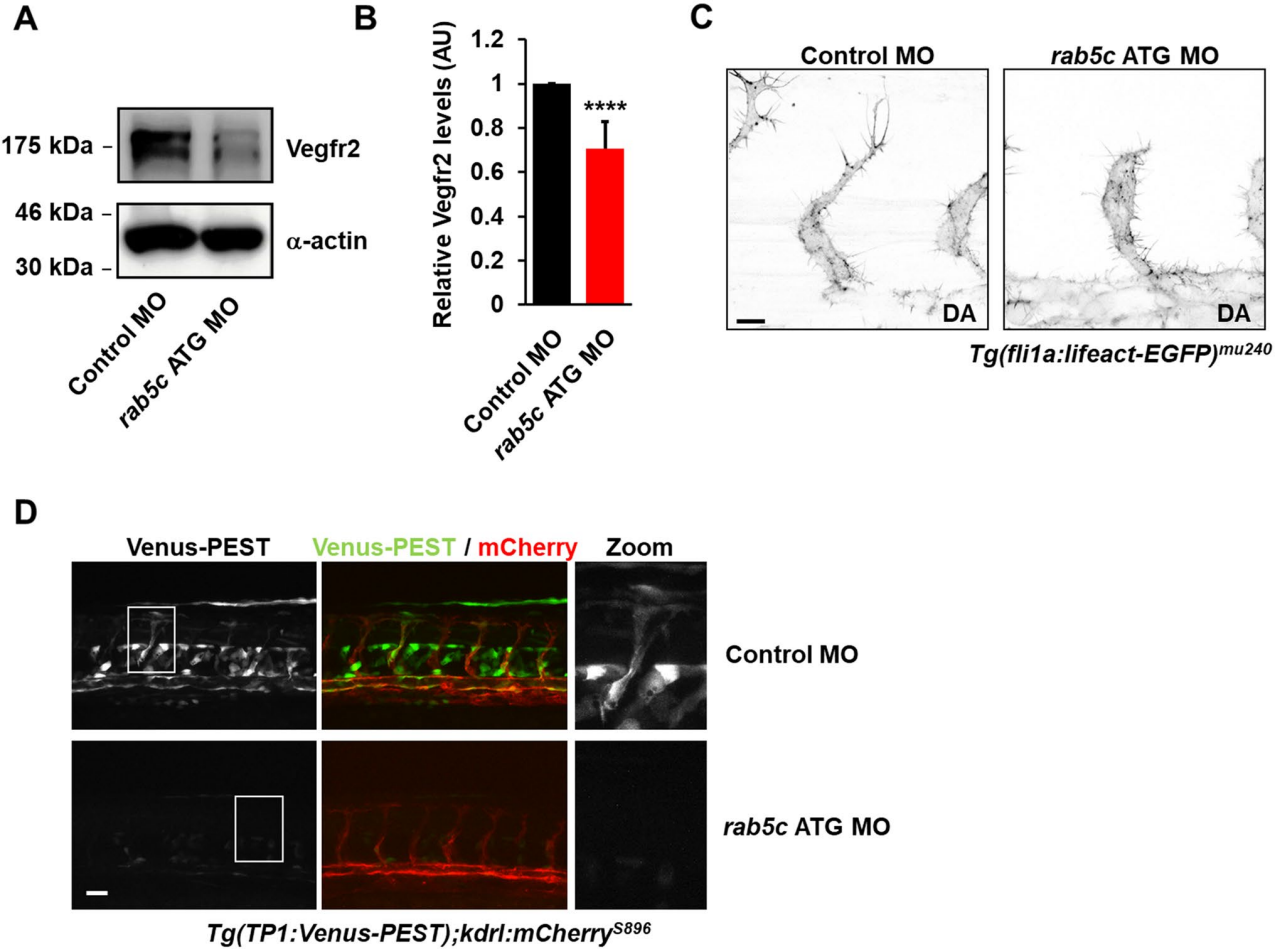
Rab5C controls Vegf and Notch signaling in vivo. **a** Western blot analysis of zebrafish lysates probed for Vegfr2 with α-actin as a loading control. A representative blot from n = 3 is shown. **b** Quantification of Western blots showing relative Vegfr2 levels (normalized to α-actin). Levels in embryos injected with Control MO were set to 1. Results are means + SEM (n = 3), 10 embryos were used per condition per experiment. AU, arbitrary units. **c** Inverted maximum projections of confocal z-stacks acquired by time-lapse microscopy in *Tg(fli1a:lifeact-EGFP)*^*mu240*^ zebrafish embryos injected with Control MO (*left*) or *rab5c* MO (*right*). Scale bar, 10 μm. DA, dorsal aorta. **d** *Tg(TP1:Venus-PEST);(kdrl-mCherry)*^*s896*^ zebrafish embryos demonstrating activation of Notch signaling in embryos injected with Control MOs (*top*), which is reduced in embryos injected with *rab5c* MOs (*bottom*). Scale bar, 20 μm

We also investigated the levels of zebrafish Vegfr2 (Kdrl) protein in embryos injected with Control MO or *rab5c* MO by Western blotting (Fig. 5a). Similar to what we observed in HUVECs, a significant reduction in Kdrl protein levels was apparent in Rab5c-depleted embryos (Fig. 5a,b). Consistently, tip cells in these embryos formed shorter filopodia, as revealed by Rab5c knockdown in *Tg(fli1a:lifeact-EGFP)*^*mu240*^ embryos, which express LifeAct-eGFP in the vasculature to visualize the actin cytoskeleton (Movies S3,S4 and Fig. 5c) [48]. Vegf-induced tip cell specification in zebrafish requires initial activation of Notch signaling in tip cells, which subsequently directs them into developing arteries [49, 50]. Since we found an impairment of tip cell morphology and behavior in Rab5c-depleted embryos, we also addressed whether Notch signaling was affected. For this purpose we used the *Tg(TP1bglob:VenusPEST)*^*s940*^ transgenic line to image the highly dynamic expression of a very short-lived Venus protein (Venus-PEST), that is driven by the TP1 promoter element and thus reports activation of Notch signaling [51]. Notch activation was visible in arteries, as well as in some tip cells and arterial ISVs (Fig. 5d), in addition to a number of non-vascular mesenchymal cells. As expected from our morphological data, a strong reduction of Venus-PEST expression in *rab5c* MO-injected embryos was observed, indicating that activation of Notch signaling was reduced and further supporting our hypothesis that Rab5c is required for efficient generation of functional tip cells (Fig. 5d).

Altogether, these data show that in line with our observations in HUVECs (Figs. 1–3), Rab5c is crucial for the maintenance of Vegfr2 levels in zebrafish, and thereby regulates tip cell formation and sprouting angiogenesis in vivo.

### The Rab5 GEF RIN2 maintains VEGFR2 levels in endothelial cells and promotes angiogenic sprouting

**Fig. 6:**
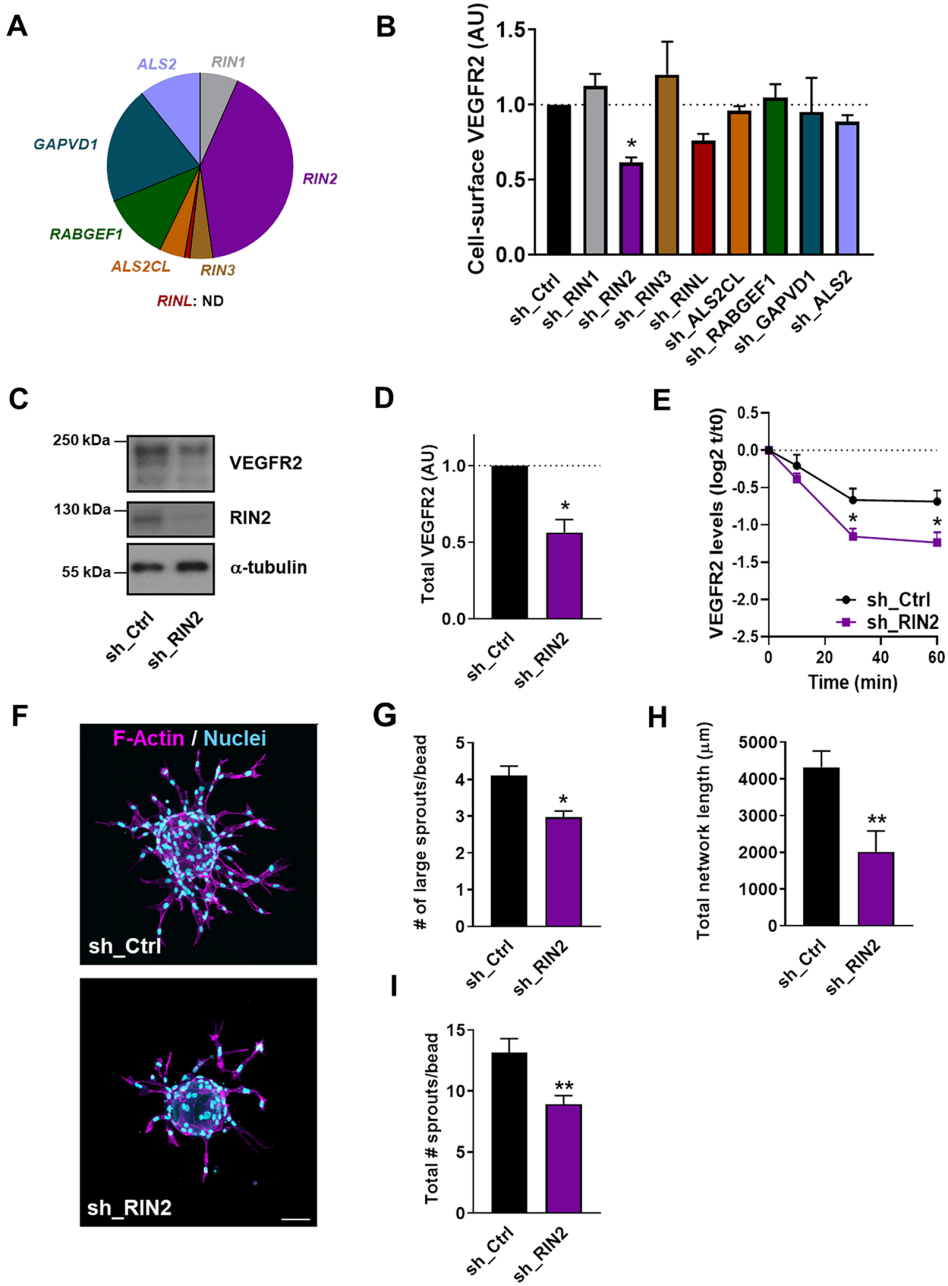
The Rab5 GEF RIN2 protects VEGFR2 from degradation and promotes angiogenic sprouting. **a** Pie diagram showing the relative mRNA levels of Rab5 GEFs in HUVECs. **b** Quantification of cell-surface levels of VEGFR2, as assessed by flow cytometry, in HUVECs transduced with shRNAs against the indicated GEFs. Values represent mean fluorescence intensities + SEM of 3–5 independent experiments, expressed relative to sh_Ctrl cells. **c** Western blot analysis of VEGFR2 and RIN2, using α-tubulin as a loading control. **d** Quantification of VEGFR2 levels from Western blots. Values represent the means + SEM of 3 individual experiments relative to sh_Ctrl. **e** Quantification of VEGFR2 degradation from Western blots in sh_Ctrl and sh_RIN2 cells that were starved overnight and subsequently either maintained in growth factor-free medium or stimulated with 50 ng/ml VEGF. Bars represent means + SEM of 3 independent experiments, expressed relative to sh_Ctrl cells at t = 0. **f** Representative images (maximum projections from confocal z-stacks) showing sprouting of sh_Ctrl and sh_RIN2 HUVECs. Staining shows F-actin (*magenta*) and nuclei (*cyan*). Scale bar, 75 μm. **g** Quantification of the average number of large sprouts/bead for sh_Ctrl and sh_RIN2 cells. Values represent means + SEM of 3 independent experiments (20 beads per experiment). Quantification of **h** total network length and **i** average total number of sprouts in sh_Ctrl (*n* = 18 beads) and sh_RIN2 cells (*n* = 16 beads). A representative experiment is shown

The previous sections have shown that activation of endothelial Rab5C is required for sprouting angiogenesis. Because Rabs are activated by GEFs, we next investigated which of the eight known GEFs for Rab5 GTPases are expressed in endothelial cells, using publicly available genome-wide mRNA expression profiles. This analysis suggested that HUVECs express at least seven different Rab5 GEFs, while *RINL* was not represented on the used arrays (Fig. 6a and Table S2). To investigate which of these GEFs is involved in the Rab5C-dependent effects on VEGFR2 and angiogenic sprouting, we silenced the expression of each individual GEF, including RINL, and assessed VEGFR2 cell-surface levels by flow cytometry. Of all investigated GEFs, only the depletion of Ras and Rab interactor (RIN) 2 significantly reduced the surface levels of VEGFR2, to the same extent as the depletion of Rab5C (Fig. 6b). Furthermore, total protein levels of VEGFR2 were also reduced by RIN2 depletion, as assessed by Western blotting (Fig. 6c, d). As in the case of Rab5C depletion, the reduction of VEGFR2 levels was associated with an increase in VEGF-induced VEGFR2 degradation (Fig. 6e). Finally, we performed a sprouting assay using RIN2-depleted cells. As expected, both the number of formed sprouts as well as the total network length were suppressed by RIN2 depletion (Fig. 6f–i).

Collectively, our data show that knockdown of RIN2 expression recapitulates the effects of Rab5C depletion, and strongly suggest that RIN2-mediated Rab5C activation prevents VEGFR2 degradation to promote sprouting angiogenesis.

### RIN2 regulates Rab5C recruitment and is required for sprouting angiogenesis in vivo

**Fig. 7:**
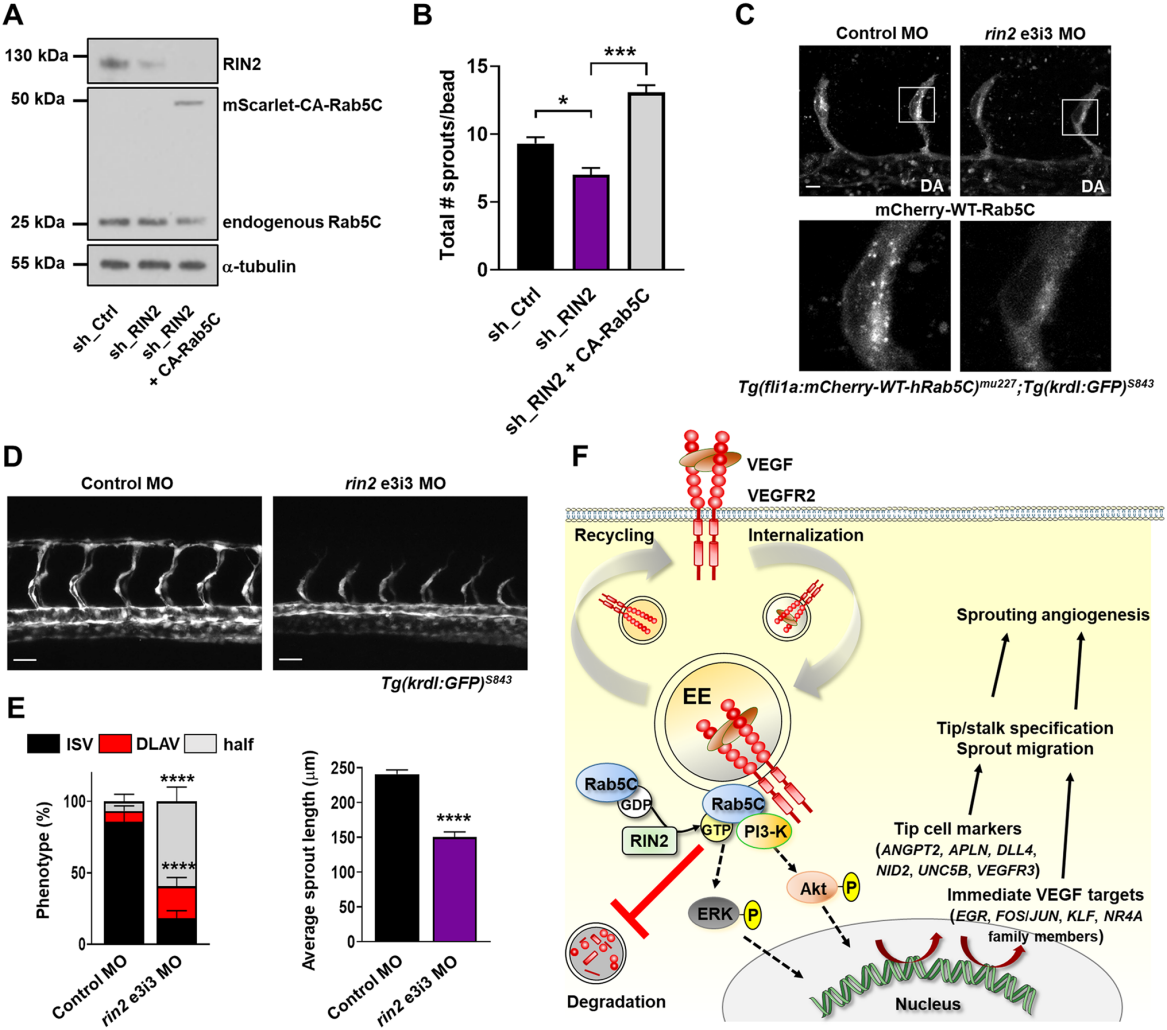
RIN2 regulates Rab5C recruitment and is required for sprouting angiogenesis in vivo. **a** Representative Western blot showing the expression of RIN2, endogenous Rab5C, and mScarlet-CA-Rab5C in HUVECs. **b** Quantification of the total number of sprouts/bead was assessed from confocal microscopy z-stacks for the indicated conditions after 24 h of sprouting. Values represent the means + SEM of *n* = 33 beads (sh_Ctrl), *n* = 35 beads (sh_RIN2), *n* = 48 beads (sh_RIN2 + CA-Rab5C), pooled from 3 independent experiments. **c** *Tg(fli1a:mCherry-WT-hRAB5C)*^*mu227*^*;Tg(kdrl:GFP)*^*s843*^ zebrafish embryos were injected with control MO (*left*) or *rin2* MO (*right*) and imaged by confocal microscopy to visualize Rab5C localization. Scale bars, 10 μm. DA, dorsal aorta. **d** Maximum projections of z-stacks obtained by confocal microscopy showing ISV formation at 32 hpf in *Tg(kdrl:GFP)*^*s843*^ zebrafish embryos injected with Control MO (*left*) or *rin2* MO (*right*). Scale bars, 40 μm. **e** Quantification of ISV development (*left*) as well as average sprout length (*right*) in zebrafish embryos injected with Control MO or *rin2* MO. Control MO: *N* = 12 embryos, *n* = 84 ISVs; *rin2* MO: *N* = 10 embryos, *n* = 72 ISVs. Indicated are statistically significant differences in DLAV and ‘half’ phenotypes compared to Control MO. **f** Model summarizing the main results of this study. Rab5C and the Rab5 GEF RIN2 protect the EE pool of VEGFR2 from VEGF-induced degradation, which sustains VEGF signaling and is required for the expression of immediate VEGF targets and tip cell genes. Together, these events regulate tip cell specification, endothelial cell migration, and sprouting angiogenesis

We next tested whether forced Rab5C activation can circumvent the requirement for RIN2 to drive sprouting. For this purpose, we generated a constitutively active (CA) mutant (Q80L) of human Rab5C, which blocks GTP hydrolysis, fused to mScarlet (Fig. S6B). Expression of this mutant into HUVECs caused enlargement of EEs, indicative of constitutive Rab5C activation (Fig. S6C) [52]. We then expressed mScarlet-CA-Rab5C into RIN2-depleted cells, which slightly lowered endogenous Rab5C expression, whereas RIN2 knockdown by itself did not affect endogenous Rab5C levels or vice versa (Fig. 7a; Fig. S6D). Importantly, expression of CA-Rab5C in RIN2-depleted HUVECs rescued the sprouting defects caused by RIN2 deficiency, and thus bypassed the requirement for RIN2 (Fig. 7b). These data confirm that 1) Rab5C activation promotes sprouting angiogenesis, and 2) the primary role of RIN2 in sprouting angiogenesis is to activate Rab5C.

To assess the effects of RIN2 knockdown on Rab5C function in vivo, we generated a splice-blocking MO (e3i3) against the zebrafish *rin2* gene (Fig. S7A,B), which was injected into *Tg(fli1a:mCherry-WT-hRAB5C)*^*mu227*^;*Tg(kdrl:GFP)*^*s843*^ zebrafish embryos to examine Rab5C localization. Depletion of zebrafish Rin2 protein resulted in a partial failure of Rab5C to localize in vesicular compartments (Fig. 7c), indicating that the recruitment of Rab5C requires Rin2 and hence, that the Rin2-Rab5c interaction is functionally conserved in zebrafish.

We also addressed the functional role of zebrafish Rin2 in sprouting angiogenesis in *Tg(kdrl:GFP)*^*s843*^ embryos. Knockdown of Rin2 resulted in impaired endothelial cell migration in the ISVs, similar to that induced by deficiency of Rab5c (Fig. 7d, e). The migration defect was again accompanied by reduced activation of Notch signaling, as analyzed by *Tp1:Venus-Pest* expression (Fig. S7C).

In summary, these results indicate that Rin2 is crucial for Rab5C activation and recruitment in endothelial cells, which regulates Vegf/Notch signaling, gene expression, tip/stalk cell specification, and sprouting angiogenesis (Fig. 7f).

## Discussion

In this study, we identify an endosomal regulatory mechanism dependent on Rab5C and RIN2 that is crucial for receptor signaling, transcriptional output, and sprouting angiogenesis. Our data indicate that RIN2 recruits Rab5C to EEs, where it prevents the degradation of internalized VEGFR2. This is required to maintain VEGFR2 levels and to sustain endosomal VEGF/VEGFR2 signaling toward PI3-K and ERK pathways. Furthermore, it is necessary for the normal expression of VEGF target genes, including various tip cell markers, as well as for the generation of functional tip cells and their migration (summarized in Fig. 7f). Our data emphasize the crucial role of endosomal trafficking for cell fate decisions, and have important implications.

First, the amplitude and duration of VEGF signaling toward PI3-K and to a lesser extent ERK is dependent on endosomal Rab5C. This observation implies that VEGF-induced PI3-K signaling occurs primarily from EEs and much less from the plasma membrane, which fits with the well-documented role of PI3-K as a direct effector of Rab5 GTPases on EEs [19]. Although it is possible that Rab5C depletion indirectly affects ERK signaling from cell-surface VEGFR2 due to the decrease of VEGFR2 levels, it seems likely that also VEGF-induced ERK signaling occurs mainly from EEs, which is consistent with the findings of others and the existence of an ERK scaffold complex on endosomes [2, 34, 53]. While VEGFR2 signaling in the absence of internalization can also occur from the plasma membrane [28, 32], our data suggest that once VEGFR2 is endocytosed, components of the endosomal machinery, such as Rab5C, are required for normal VEGF signaling.

Second, our findings show that endosomal maintenance of VEGFR2 levels is required for transcriptional responses that determine cell fate. The time-frame in which most of the VEGFR2 degradation occurs in the absence of Rab5C, is also the time-frame that is necessary for VEGF signaling and the induction of immediate VEGF-responsive genes. Because Rab5C depletion leads to significant and quantitative differences in the expression of most VEGF-induced genes, our observations imply that accelerated VEGFR2 degradation results in disrupted VEGF-induced gene expression. Thus, the magnitude of VEGF-induced gene expression is closely related to VEGFR2 levels, and stabilization of endosomal VEGFR2 levels by Rab5C ensures the build-up of a sufficiently robust VEGF signal needed to surpass the threshold for transcription. Similar mechanisms likely regulate the duration of signaling of other receptors in different contexts, which is particularly interesting given the frequent overexpression of several Rab GTPases in cancer and many other diseases [54].

Third, the reduction in VEGFR2 levels induced by depletion of Rab5C is functionally and physiologically crucial, since we observe functional defects in tip cell behavior and angiogenic sprouting, both in vitro and in vivo. Tip cells are characterized by high VEGFR2 levels and thus high VEGF signaling, leading to the expression of tip cell markers [5–7]. Because VEGFR2 is downregulated in Rab5C-depleted cells whereas VEGFR1 levels are not affected, the VEGFR1/VEGFR2 balance is disturbed which confers a competitive disadvantage to develop tip cell properties [11–13]. Indeed, the expression of various tip cell genes was decreased by Rab5C depletion, and tip cell formation was impaired in mosaic sprouting assays in vitro. While it is known that balanced VEGFR2 signaling regulates tip cells, we show that this crucially depends on endosomal regulation of VEGFR2 in a Rab5C-dependent manner, and that Rab5C deficiency is sufficient to affect all aspects of VEGF-driven tip cell responses, namely tip cell specification, function, and characteristics. Interestingly, out of eight different Rab5 GEFs, only the depletion of RIN2 specifically recapitulates our main findings in Rab5C-depleted cells. Although RIN2 may activate several Rabs and Rab5C might use multiple GEFs for all of its functions, our data indicate that RIN2 is the most important Rab5 GEF for the effects of RabC on VEGFR2 traffic and sprouting angiogenesis. These results nicely complement previous work on RIN2 in endothelial cells, where RIN2 was found to be important for several processes in endothelial cells including tube formation [55]. In line with previous reports of a high molecular and functional conservation of zebrafish and mammalian angiogenesis [46, 47], we could show full conservation of the Rin2/Rab5c-dependent Vegfr2-signaling axis in zebrafish, allowing us to analyze tip cell behavior and sprouting angiogenesis in response to Rin2/Rab5c-driven endosomal Vegfr2 signaling in vivo. In zebrafish embryos, depletion of Rab5c or Rin2 attenuated Vegf-induced activation of Notch signaling in the tip cells, filopodia formation, and endothelial cell migration, thus affecting key aspects of sprouting angiogenesis. Together, our data suggest that RIN2/Rab5C control a feedforward mechanism whereby inhibition of VEGFR2 degradation permits sprouting angiogenesis, by sustaining VEGF signaling and the expression of VEGF target genes. The endolysosomal machinery is therefore an important driver of the dynamic regulation of tip and stalk cells, and likely contributes strongly to their rapid and frequent interconversion observed during sprouting [56].

To date, few factors are known that protect VEGFR2 from degradation. Depletion of Numb or Rab GTPase-binding effector protein-2 (RABEP-2) also enhances VEGFR2 degradation, resulting in lower VEGFR2 levels and reduced VEGF signaling [27, 33]. Interestingly, genetic deletion of RABEP-2 in mice disturbs arteriogenesis but not angiogenesis, while the deletion of Numb induces angiogenic defects [27, 57]. It remains to be determined how different proteins along trafficking pathways can mediate such very diverse outcomes of VEGFR2 signaling. Future work should further establish how the fate of internalized VEGFR2 is linked to the transcriptional output of VEGF signaling and the dynamic regulation of endothelial cell phenotypes, as well as map the underlying molecular machinery along trafficking routes.

## Materials and methods

### Antibodies, plasmids and other materials

The following antibodies were used: anti-VEGFR2 (R&D Systems, AF357), anti-α-tubulin (Sigma-Aldrich, T6199), anti-AKT (Cell signaling technology, #9272), anti-p(S473)AKT (Cell signaling technology, #4060S), anti-VEGFR1 (Abcam, ab32152), anti-p(Y204)ERK-1/2 (Santa Cruz Biotechnology, sc-7383), anti-ERK-1/2 (Santa Cruz Biotechnology, sc-153), and anti-RIN2 (Sigma-Aldrich, HPA034641). HRP-conjugated secondary antibodies (#P0447, #P0399 and #P0449) were from DAKO, Alexa Fluor-conjugated secondary antibodies (A21445, A21200, and A21467) were from Thermo Fisher. Recombinant human VEGF-A (VEGF165) was purchased from R&D Systems (293-VE), fibronectin (#F1141) and thrombin (#T1063) were from Sigma-Aldrich, human fibrinogen (Haemocomplettan P, B02BB01) was from CSL Behring. Bafilomycin (#tlrl-baf1) was from InvivoGen, Leupeptin (#4041) and Pepstatin (#4397) were from PeptaNova, Hoechst 33,342 (#H3570), phalloidin (#T7471), and CellTracker Fluorescent Probes (CMFDA green, #C2925 and CMTPX red, #C34552) were from Thermo Fisher Scientific. All used primers were purchased from Integrated DNA Technologies, Inc. (Table S3). The human WT and DN *RAB5C* open reading frames (ORFs) were synthesized, cloned into *pDONR221*, and sequence-verified by Thermo Fisher Scientific for gateway cloning. For the zebrafish experiments, the *RAB5C* ORFs were cloned into a modified version of *pTol2-fli1ep:CherryDest* (a kind gift from Nathan Lawson; Addgene plasmid #73,493). To generate this destination vector, the mCherry sequence was PCR-amplified without stop codon using oligos extended with restriction sites (fwd: 5′-GACGATGCTAGCGCCACCATGGTGAGCAAGGGCGAG-3′ rev: 5′-GACGATTACGTAGATATCCTTGTACAGCTCGTCCATG-3′). This product was cloned into *pTol2-fli1ep:CherryDest* using the *Nhe*I and *Sna*BI restriction sites. Next, a Gateway Reading Frame Cassette A containing the appropriate attR1/2 sites was cloned into the *Eco*RV site, 3′ of the mCherry ORF, creating *pTol2-fli1ep:CherryDestV2*. Finally, the human WT and DN *RAB5C* ORFs in *pDONR221* were cloned into *pTol2-fli1ep:CherryDestV2* using LR Clonase II enzyme mix (Invitrogen), creating the 4 *pTol2-fli1ep:Cherry-RAB5* plasmids. For lentiviral transduction of the *RAB5* ORFs into HUVECs, we modified *pLenti6.3/V5-Dest* (Invitrogen) by removing an attR1/2 cassette by digestion with *Eco*RI and *Eco*RV, and introducing an *Nhe*I site using 2 oligos (5′-AATTCGCTAGCGACGACGAT-3′ and 5′-ATCGTCGTCGCTAGCG-3′). Next, the *Nhe*I/*Sna*BI fragment from *pTol2-fli1ep:Cherry-RAB* was cloned into the *Nhe*I/*Eco*RV sites of *pLenti6.3/V5-Dest*. To generate the mScarlet-Rab5C-Q80L construct, site-directed mutagenesis was performed on an mScarlet-Rab5C-wt construct using primer 5′-GTTTGAGATCTGGGACACAGCTGGA**CTG**GAGCGGTATCACAGC-3′ and its reverse complement, in which CAG, coding for Q (Glutamine) was replaced with CTG coding for L (Leucine). All cloned plasmids used in experiments were sequenced with at least double coverage.

### Cell culture, lentiviral transduction, and RNA interference

Primary HUVECs pooled from 3 to 5 individual donors (Lonza, C2519A) were cultured in endothelial growth medium-2 (EGM-2; Promocell, C-22011) supplemented with 2 mM L-glutamine (Sigma-Aldrich) and 100 U/ml penicillin and 100 μg/ml streptomycin (Sigma-Aldrich). Cell culture flasks and dishes were coated with 0.1% (w/v) gelatin (Sigma-Aldrich) unless stated otherwise. HUVECs were used between passages 3 and 6. To target Rab5C or the indicated GEFs, we used shRNAs cloned into pLKO.1 (3–9 per gene, Table S4) from the TRC Mission Library (a generous gift from Roderick Beijersbergen, The Netherlands Cancer Institute, Amsterdam). Human embryonic kidney (HEK) 293 T cells (ATCC, CRL-3216) were maintained in Dulbecco’s modified Eagle medium (DMEM) (Thermo Fisher Scientific) containing 4.5 g/l D-glucose, 2 mM L-glutamine, 10% (v/v) fetal bovine serum (Bodinco), 1 mM sodium pyruvate (Thermo Fisher Scientific), and 100 U/ml penicillin and 100 μg/ml streptomycin. To produce lentiviral particles containing shRNAs, HEK293T cells were transfected using *Trans*IT-LT1 transfection reagent (Mirus Bio) according to the manufacturer’s protocol. Supernatant was harvested 48 and 72 h after transfection, centrifuged, filtered over a 0.45 μm pore filter, aliquoted and stored at -80ºC. HUVECs were lentivirally transduced with either a pool of shRNAs, or with a scrambled sequence in pLKO.1 as a negative control. Positive cells were selected during 3 days using 1 μg/ml puromycin (Sigma-Aldrich).

### qPCR

For analysis of relative mRNA expression levels in transduced HUVECs, total RNA was isolated using the RNeasy kit (Qiagen) according to the manufacturer’s instructions. After reverse transcription to cDNA using the SuperScript III First-Strand Synthesis System (Thermo Fisher Scientific), qPCR was performed with the SensiFAST SYBR No-ROX kit (Bioline) and the indicated primers (Table S3), either on a LightCycler PCR system (Roche) or a StepOnePlus system (Applied Biosystems). Duplicate reactions were performed for each gene and expression was normalized to that of β-actin.

### Flow cytometry

HUVECs were treated as indicated, detached with accutase (Sigma-Aldrich) for 2 min, and washed in 2% FCS in PBS. Cells were stained with primary antibodies for 1 h on ice, washed twice, followed by incubation with secondary antibodies for 45 min on ice and washed twice. Cells were analyzed on a Canto-II flow cytometer (BD Immunocytometry Systems) equipped with FACSDiva software.

### Bio-informatic analysis of mRNA expression

Expression of Rab5 GEFs in HUVECs (Table S2) was determined by analysis of Affymetrix U133 Plus 2.0 genome-wide mRNA expression profiles in the public domain using the NCBI Gene Expression Omnibus (GEO: GSE7307) website [58, 59]. GEO was first searched for studies on low-passage, non-recombinant, non-stimulated HUVECs with data normalization using the MAS5.0 algorithm (Affymetrix Inc., Santa Barbara, CA). This resulted in a total of 11 published studies comprising 29 separate arrays, 17 of which were listed with present call analysis, that were queried for the expression of all known human Rab5 GEFs [20]. RINL was not represented on the Affymetrix arrays and was not further analyzed. Array data were analyzed as described using R2; an in-house developed Affymetrix analysis and visualization platform (http://r2.amc.nl). Probes were ranked depending on high expression values and widespread expression (% of samples with significant expression for that gene) in the data collections tested.

### Western blotting

Cells were washed in ice-cold PBS and lysed on ice in NP40 lysis buffer (50 mM Tris–HCl, pH 7.4, 100 mM NaCl, 10 mM MgCl_2_, 1% NP-40, 10% Glycerol), supplemented with protease inhibitor cocktail (Sigma-Aldrich). Cell lysates were cleared by centrifugation, heated at 95ºC in SDS sample buffer (50 mM Tris–HCl pH 6.8, 2% SDS, 10% glycerol, 1% β-mercaptoethanol, 12.5 mM EDTA, 0.02% bromophenol blue), and proteins were resolved by SDS-PAGE. Thereafter, proteins were transferred to nitrocellulose membranes (GE Healthcare Amersham) and aspecific binding was blocked using 5% (w/v) milk (Campina) in TBST (150 mM NaCl, 10 mM Tris, 0.1% Tween 20, pH 8.0) for 30 min. Immunoblotting was performed with the indicated antibodies by incubation with primary antibodies o/n at 4ºC and with secondary antibodies for 1 h at RT. Membranes were washed 3 × with TBST after each step. Proteins were visualized using ECL chemiluminescence reagent (Thermo Fisher Scientific), light sensitive films (Fuji Film) and a film processor (Konica Minolta, SRX-101A). Quantification of bands was performed by densitometry using ImageJ. Band intensity of the protein of interest was corrected to that of α-tubulin.

### Degradation and signaling experiments

For VEGF signaling and VEGFR2 degradation assays, HUVECs in 6-well plates were grown to confluence. Then, cells were starved overnight and subsequently either left untreated or stimulated with 50 ng/ml VEGF for the indicated time-points, whereafter the cells were processed for Western blotting. Inhibitors were used as indicated.

### Sprouting assays

Sprouting assays were performed according to previously established protocols with minor modifications [42, 43]. In brief, HUVECs were incubated with collagen-coated microcarrier beads (Sigma-Aldrich, C3275). The next day, the beads were detached by washing and transferred to 48-well plates containing fibrinogen in PBS (2 mg/ml) supplemented with 0.15 U/ml aprotinin and 6.25 U/ml thrombin. EGM-2 medium was added on top of the gels. Beads were imaged by time-lapse microscopy on a Widefield system using a 10 × dry lens objective (Carl Zeiss MicroImaging, Inc.). Images were recorded at 15-min intervals during 48 h. For each condition, 20 beads were analyzed per experiment. Sprouting was assessed as the average number of large (length of sprout > than the bead diameter) sprouts per bead. Alternatively, beads were embedded in fibrin gels in ‘half-area’ glass-bottom 96-well imaging plates (Corning, #4580). After 48 h, beads were fixed, stained with Hoechst and phalloidin, and visualized by generating z-stacks on a confocal microscope. Maximum projections of z-stacks were created using Fiji/ImageJ (version 1.52e), de-speckled once, and the contrast was enhanced with 0.3% and analyzed using the *Angiogenesis* plugin [60]. The phalloidin staining was used for correct segmentation of the sprouts. For the sprouts stained with CellTracker, the number of tip cells per condition was counted and normalized to the total number of cells in that condition.

### Zebrafish husbandry and strains

Zebrafish (*Danio rerio*) husbandry and embryo maintenance were carried out under standard conditions at 28.5 ℃ [61]. Embryonic developmental stages were determined as described [62]. Transgenic lines used in this study were *Tg(kdrl:GFP)*^*s843*^, *Tg(kdrl:Hras-mCherry)*^*s896*^, *Tg(TP1bglob:VenusPEST)*^*s940*^, *Tg(fli1a:lifeact-EGFP)*^*mu240*^ [48, 51, 63, 64]. All animal experiments were performed in compliance with the relevant laws and institutional guidelines, were approved by local animal ethics committees and were conducted at the University of Münster and the MPI for Molecular Biomedicine with permissions granted by the Landesamt für Natur, Umwelt und Verbraucherschutz (LANUV) of North Rhine-Westphalia.

### Generation of transgenic and *rab5c* mutant lines

The transgenic lines *Tg(fli1a:mCherry-WT-hRAB5C)*^*mu227*^ and *Tg(fli1a:mCherry-DN-hRAB5C)*^*mu228*^ zebrafish lines were newly generated using Tol2-mediated DNA integration [65]. The *rab5c*^*mu229*^ mutation was newly generated by CRISPR/Cas9. Annealed template oligonucleotides were transcribed into gRNAs using MEGAshortscript T7 Kit (Ambion) as described [66]. The following oligos were used for gRNA generation: *rab5c* 5′-AAAGCACCGACTCGGTGCCACTTTTTCAAGTTGATAACGGACTAGCCTTATTTTAACTTGCTATTTCTAGCTCTAAAACCCGTCGGGAACAAAATCTGCCTATAGTGAGTCGTATTACGC-3′ (*rab5c* target sequence: 5′-GCAGATTTTGTTCCCGACGGGGG). A total of 1 nl of 300 ng/ml gRNA and 3.22 μg/μl Cas9 NLS protein (M0646T, New England Biolabs) were injected at the single-cell stage. Efficiency of the CRISPR-targeting as well as genotyping were performed by PCR, using fwd 5′-GCCTCTCCATCCTTTTCTCA-3′ and rev 5′-TCACGAACACACTCCAGCTC-3′ and subsequent restriction enzyme digest with *Hpy*99I.

### MOs and microinjections

For MO-mediated gene knockdown, embryos were injected at the one-cell stage with 3 ng control MO (Gene Tools), 5′-CCTCTTACCTCAGTTACAATTTATA-3′, 3 ng *rab5c* MO, 5′-CGCTGGTCCACCTCGCCCCGCCATG-3′ 3 ng *rin2* MO, 5′-GCAGTGTGTTTTAACTCTCACCTTA-3′. Sequence of the *rab5c* ATG MO was as previously described [37]. The *rab5c* and *rin2* splice-site MOs were generated as shown (Fig. S6A,S8A), and validated by RT-PCR. For mosaic overexpression of mCherry-WT-Rab5C or mCherry-DN-Rab5C, embryos were injected at the single-cell stage with 100 pg plasmid and 250 pg Tol2 mRNA per embryo. Effects on vascular development were analyzed at 30–32 hpf.

### RT-PCR and Western blotting in zebrafish

Total RNAs were prepared from 36 hpf embryos using TRIzol reagent (Invitrogen), and reverse-transcribed by random hexamer primers using Superscript III (Invitrogen) according to the manufacturer’s instruction. PCR was performed using gene-specific primers as below. Efficiency of the knockdown was assessed by PCR using the primers fwd: AGAGCTGCGTACATCCAACC and rev: TGCAAGCCAAAATTCAAAGA.

After removing chorionic membrane and yolk sac, embryos injected with either control MO or *rab5c* MO were directly lysed in 1 × SDS sample buffer, and subjected to Western blot analysis with anti-Kdrl antibody (Kerafast, ES1003) and anti-alpha-Actin antibody (Santa Cruz Biotechnology, sc-47778) as described previously [67].

### Confocal microscopy

HUVECs on glass coverslips were fixed with 4% paraformaldehyde (Merck) in PBS containing 1 mM CaCl_2_ and 0.5 mM MgCl_2_ (PBS + +) for 10 min, and permeabilized with 0.4% Triton X-100 (Sigma-Aldrich) in PBS +  + for 5 min. Aspecific antibody binding was prevented by blocking with 2% BSA Fraction V (Sigma-Aldrich) in PBS +  + for 15 min. Following incubation with the indicated primary antibodies, coverslips were washed with 0.5% BSA Fraction V in PBS +  + and antibody binding was visualized using secondary antibodies. Coverslips were washed and subsequently mounted in 10% Mowiol, 2.5% Dabco, 25% glycerol, pH 8.5. Image acquisition was performed on a Leica SP8 confocal microscope using a 63 × oil immersion objective, after which images were processed using Leica Application Suite X and Fiji/ImageJ (version 1.52e) software.

Sprouting was visualized using a 25 × long-distance water objective on a Leica SP5 confocal set-up. z-stacks were created of 1.5 μm per slice and the different channels were sequentially scanned between stacks.

Confocal microscopy of zebrafish was performed using an LSM780 (Carl Zeiss Microscopy GmbH; objective lens: Plan Achromat 20/0.8, 20x; LD C-Apochromat 40x/1.1 W Korr M27). Living zebrafish embryos were embedded in 1% agarose (dissolved in E3 medium containing Tricaine) and analyzed using Imaris 9 software (Bitplane) as previously described [68].

For time-lapse analysis in zebrafish, a temperature of 28.5 °C was maintained using a heating chamber. Images were collected every 6 min during 1 h (for imaging of filopodia) or every 15 min during 6 h (for imaging of ISV sprouting). Assembly of confocal stacks and time-lapse movies was conducted using Imaris 8/9 software (Bitplane).

### mRNA sequencing and analysis

sh_Ctrl, sh_Rab5A or sh_Rab5C cells were seeded in 10 cm dishes and grown to confluence. Cells were starved overnight, whereafter they were either left untreated or stimulated with 50 ng/ml VEGF for 1 h or 4 h. Subsequently, cells were lysed using RLT buffer (Qiagen) with 1% β-mercapto-ethanol and stored at −80 °C until analysis. RNA-seq libraries were prepared from samples of 2 independent experiments with the mRNA KAPA HyperPrep Kit (Illumina) using Truseq Illumina LT adapters. Sequencing was performed on an Illumina Hiseq4000 system (single-read 50 bp), 10 samples per lane.

Reads were subjected to quality control (FastQC, dupRadar, Picard Tools), trimmed using Trimmomatic v0.36, and aligned to the hg38 genome using HISAT2 v2.1.0. Counts were obtained with HTSeq v0.11.0. Statistical analyses were performed using the edgeR and limma/voom packages [69, 70]. Genes with no counts in any of the samples were removed while genes with more than 2 reads in at least 3 of the samples were kept. Count data were transformed to log2-counts per million (logCPM), normalized by applying the trimmed mean of M-values method and precision-weighted using voom. Differential expression was assessed using an empirical Bayes’ moderated t-test within limma’s linear model framework including the precision weights. Resulting *p* values were corrected for multiple testing using the Benjamini–Hochberg false discovery rate. Additional gene annotation was retrieved from Ensembl (release 94) using the biomaRt/Bioconductor package. Geneset enrichment was performed using CAMERA with a preset value of 0.01 for the inter-gene correlation using collections H, C1, C2, C3, C5, C6, and C7 retrieved from the Molecular Signatures Database (MSigDB v6.2; Entrez Gene ID version), complemented with a user-defined geneset containing the genes constituting the observed VEGF transcriptome. Data analysis were performed using R (v3.5.2) and Bioconductor (v3.8). Sequencing data have been made available in the GEO under accession number: GSE134947.

### Statistical analysis

Statistical analysis was performed using one-way ANOVA for multiple comparisons and unpaired *t*-tests for comparisons between two conditions, unless stated otherwise. Throughout the paper, statistically significant differences are indicated by * (*p* < 0.05), ** (*p* < 0.01), *** (*p* < 0.001), or **** (*p* < 0.0001).

## Supplementary Information

Below is the link to the electronic supplementary material.

Electronic supplementary material 1 (TIF 83 kb)

Electronic supplementary material 2 (TIF 1306 kb)

Electronic supplementary material 3 (TIF 773 kb)

Electronic supplementary material 4 (TIF 1159 kb)

Electronic supplementary material 5 (TIF 813 kb)

Electronic supplementary material 6 (TIF 968 kb)

Electronic supplementary material 7 (TIF 707 kb)

Electronic supplementary material 8 (DOCX 34 kb)

Electronic supplementary material 9 (MP4 16027 kb)

Electronic supplementary material 10 (MP4 29646 kb)

Electronic supplementary material 11 (MP4 7774 kb)

Electronic supplementary material 12 (MP4 4913 kb)

Electronic supplementary material 13 (XLS 92 kb)

## Abbreviations

ALS2: Alsin
ALS2CL: ALS2 C-terminal-like protein
ANGPT2: Angiopoietin-2
APLN: Apelin
CA: Constitutively active
Ctrl: Control
DLAV: Dorsal longitudinal anastomotic vessel
DLL4: Delta Like ligand-4
DN: Dominant-Negative
EE: Early Endosome
EEA-1: Early Endosomal Autoantigen-1
ERK: Extracellular-signal Regulated Kinase
FDR: False discovery rate
GAPVD1: GTPase Activating Protein And VPS9 Domains 1
GEF: Guanine Exchange Factor
GEO: Gene Expression Omnibus
GFP: Green fluorescent protein
HUVEC: Human Umbilical Vein Endothelial Cells
hpf: Hours post-fertilization
ISV: Intersegmental vessel
MO: Morpholino
NID2: Nidogen-2
NR4A: Nuclear Receptor Subfamily 4 Group A
NRP2: Neuropilin-2
ORF: Open reading frame
PDGFB: Platelet-Derived Growth Factor B
PI3-K: Phosphatidylinositol 3-kinase
qPCR: Quantitative PCR
RABEP-2: Rab GTPase-binding effector protein 2
RIN: Ras and Rab Interactor
RINL: Ras and Rab INteractor-Like
sh: Short hairpin
TGN: Trans-Golgi network
VE-cadherin: Vascular Endothelial-cadherin
VEGF: Vascular Endothelial Growth Factor
VEGFR: Vascular Endothelial Growth Factor Receptor
WT: Wild-type
