# Supplementary figures and images for: The endosomal RIN2/Rab5C machinery prevents VEGFR2 degradation to control gene expression and tip cell identity during angiogenesis
Source: Angiogenesis. 2021 May 13;24(3):695–714. doi: 10.1007/s10456-021-09788-4 (PMC8292304; doi:10.1007/s10456-021-09788-4)

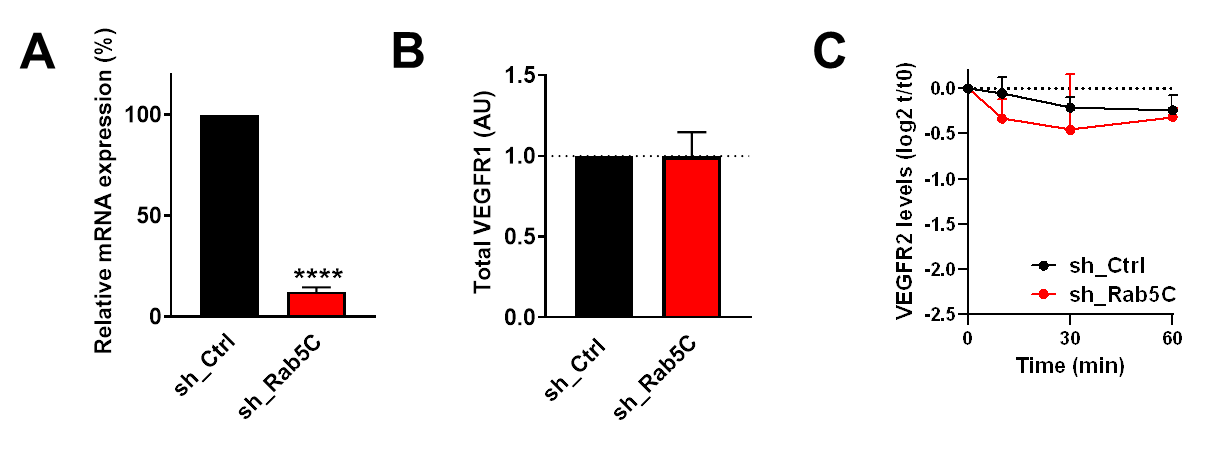

Supplement: Supplementary file 1 — Electronic supplementary material 1 (TIF 83 kb) [file 10456_2021_9788_MOESM1_ESM.tif]

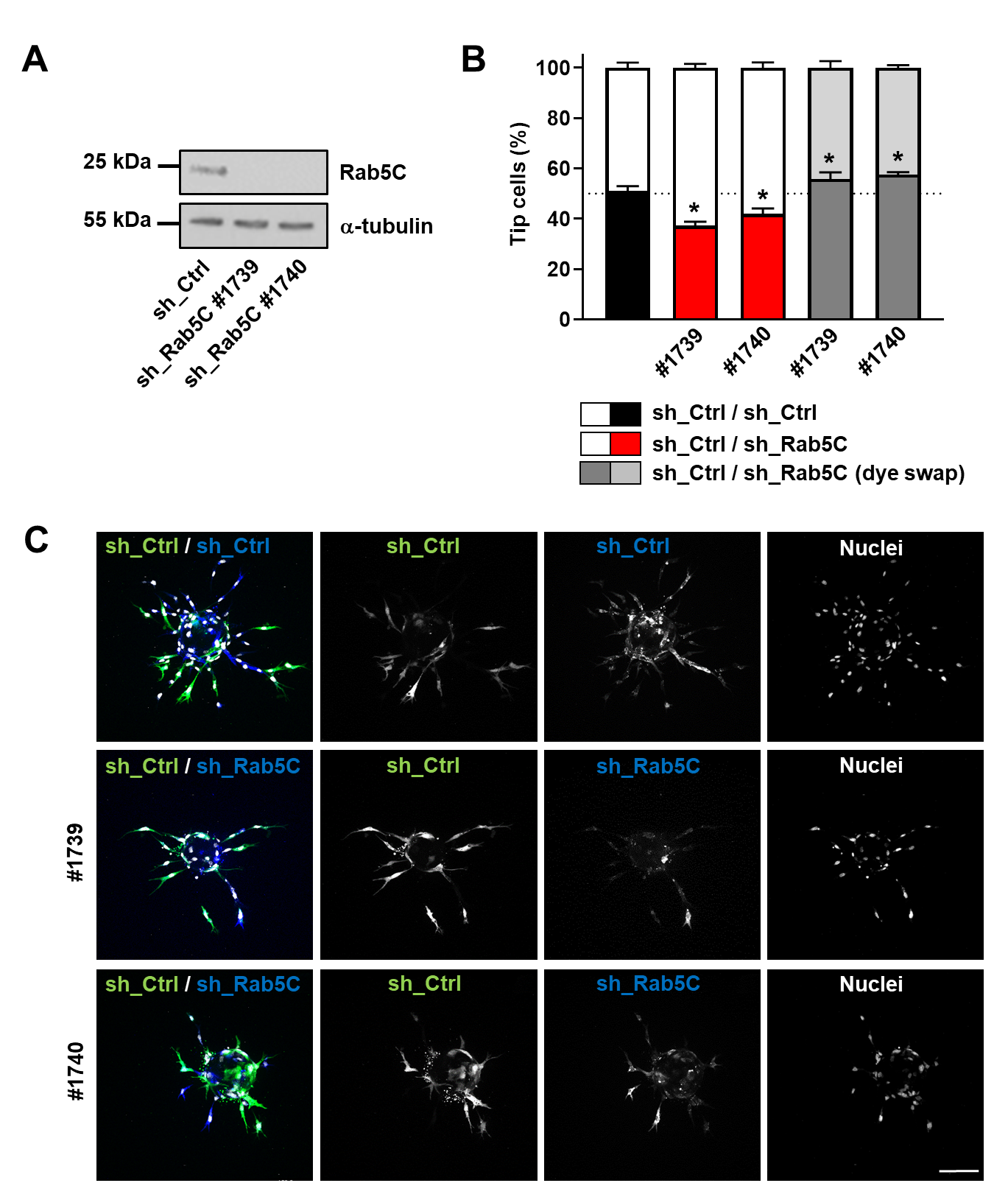

Supplement: Supplementary file 2 — Electronic supplementary material 2 (TIF 1306 kb) [file 10456_2021_9788_MOESM2_ESM.tif]

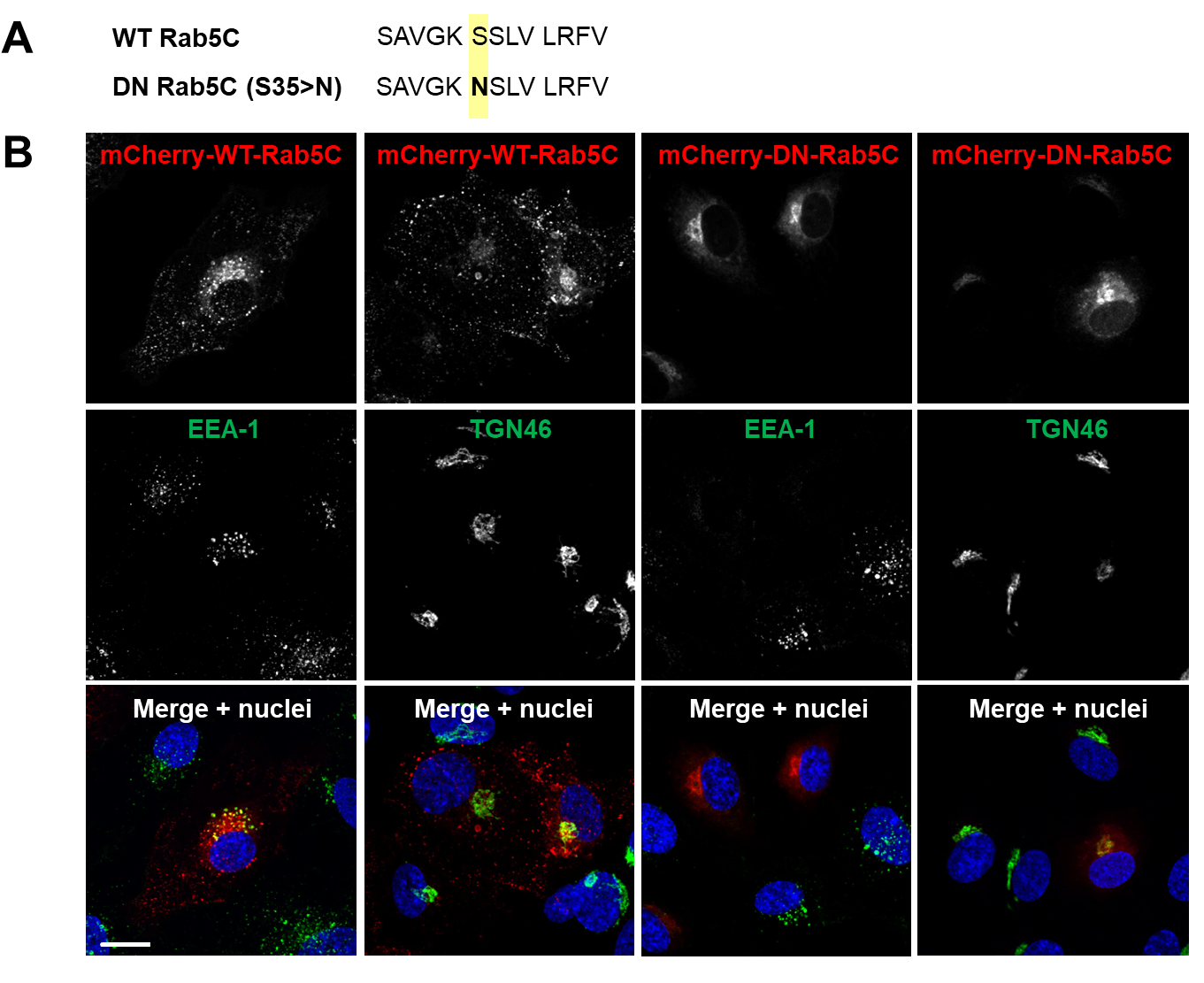

Supplement: Supplementary file 3 — Electronic supplementary material 3 (TIF 773 kb) [file 10456_2021_9788_MOESM3_ESM.tif]

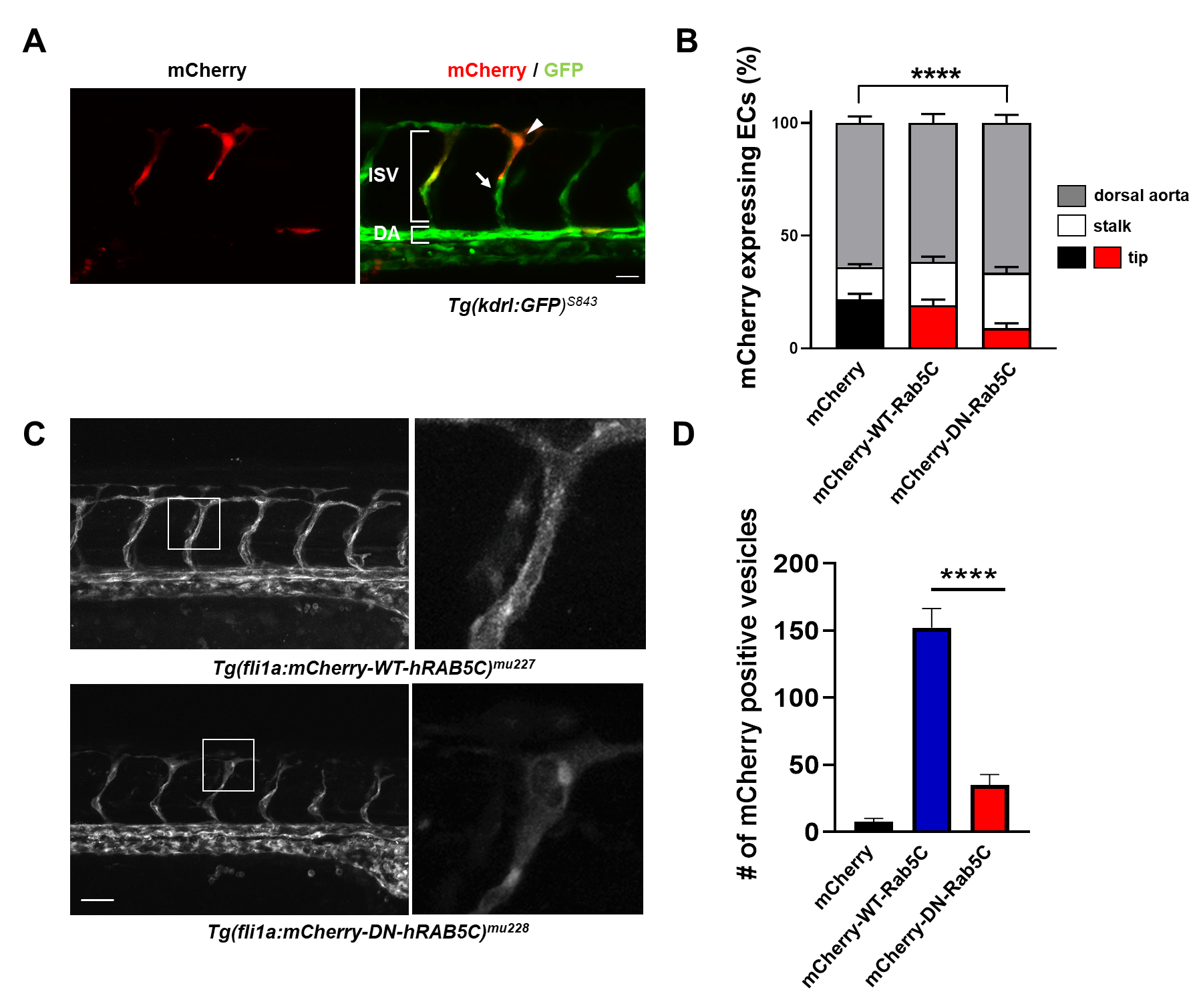

Supplement: Supplementary file 4 — Electronic supplementary material 4 (TIF 1159 kb) [file 10456_2021_9788_MOESM4_ESM.tif]

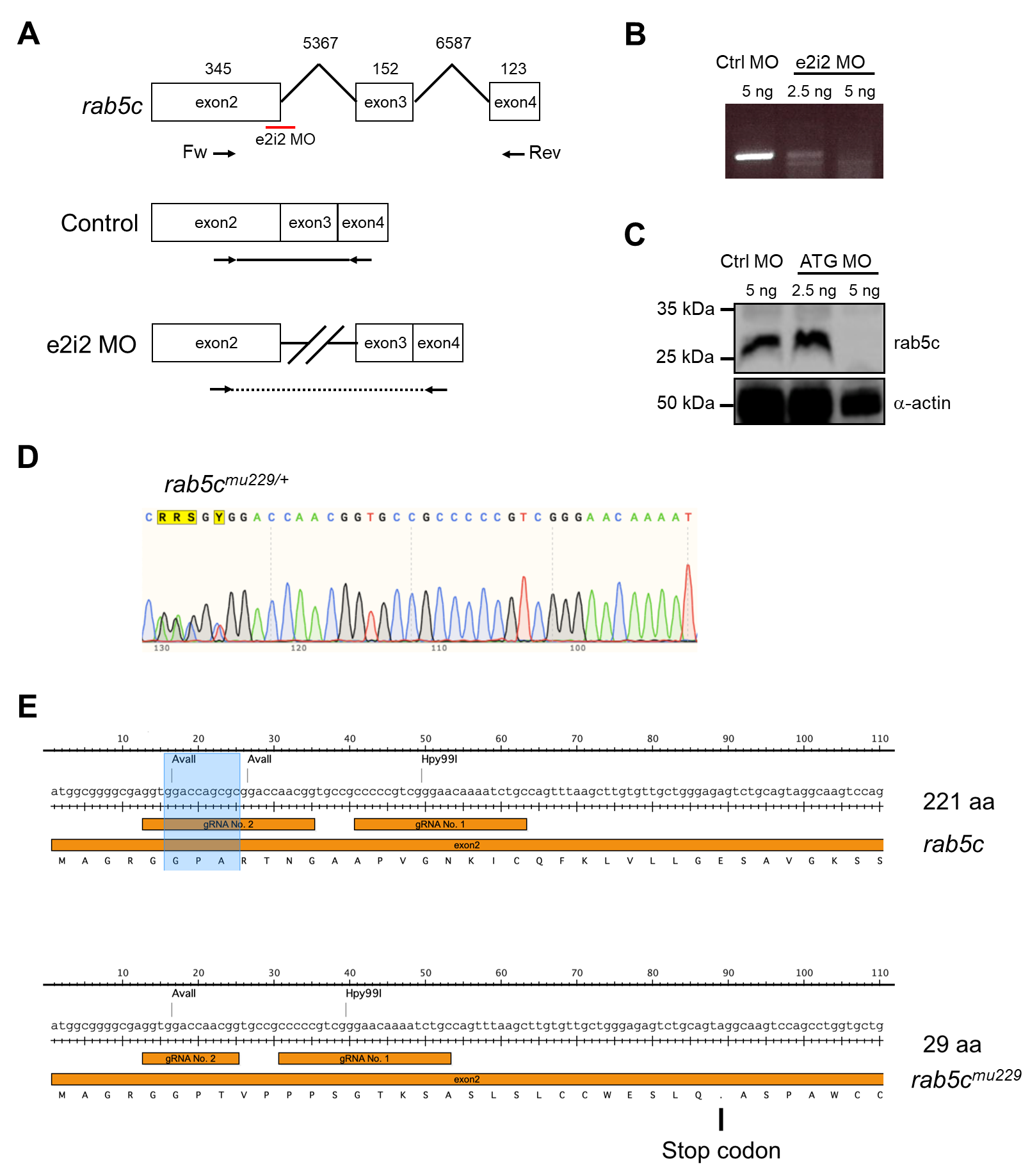

Supplement: Supplementary file 5 — Electronic supplementary material 5 (TIF 813 kb) [file 10456_2021_9788_MOESM5_ESM.tif]

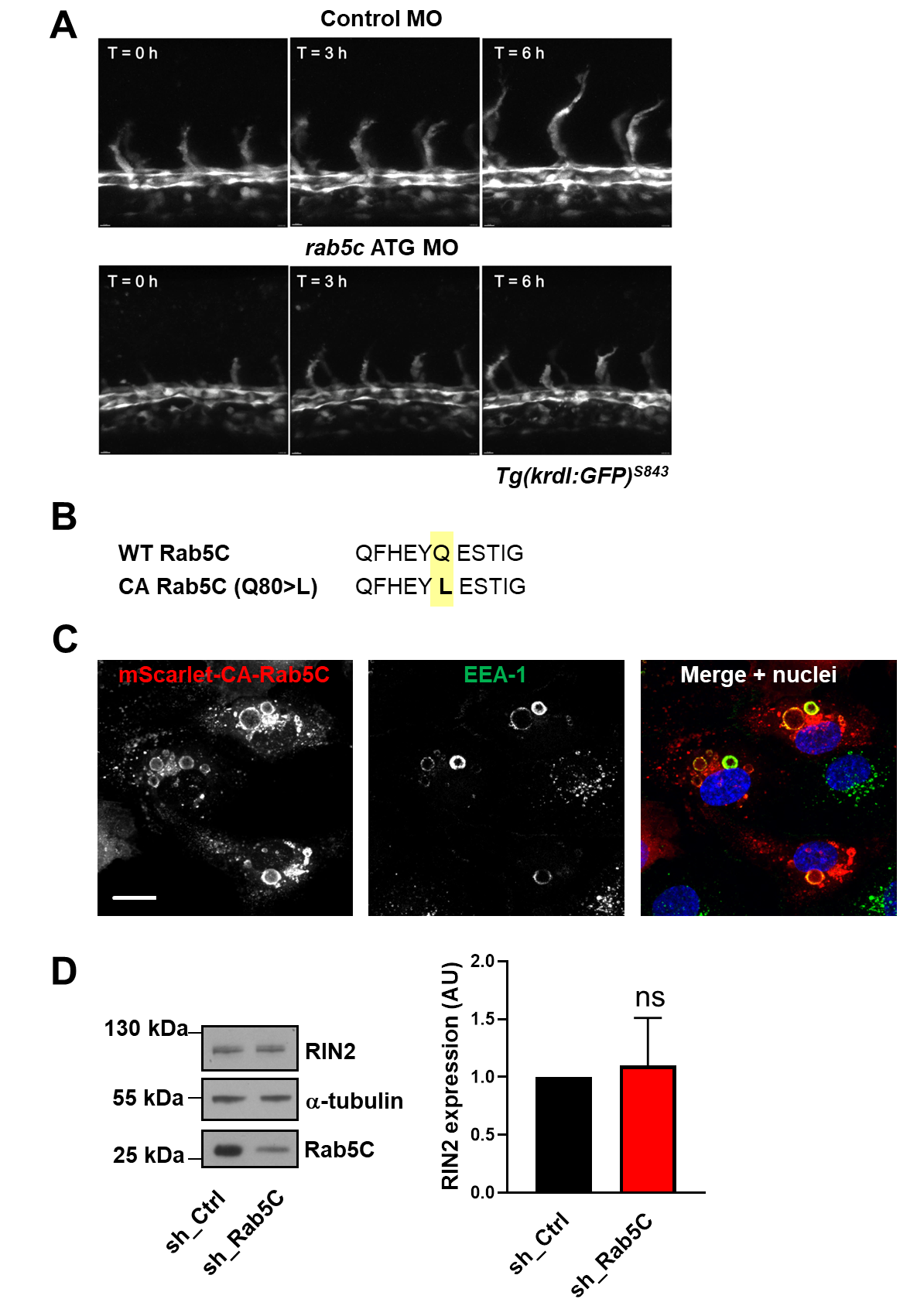

Supplement: Supplementary file 6 — Electronic supplementary material 6 (TIF 968 kb) [file 10456_2021_9788_MOESM6_ESM.tif]

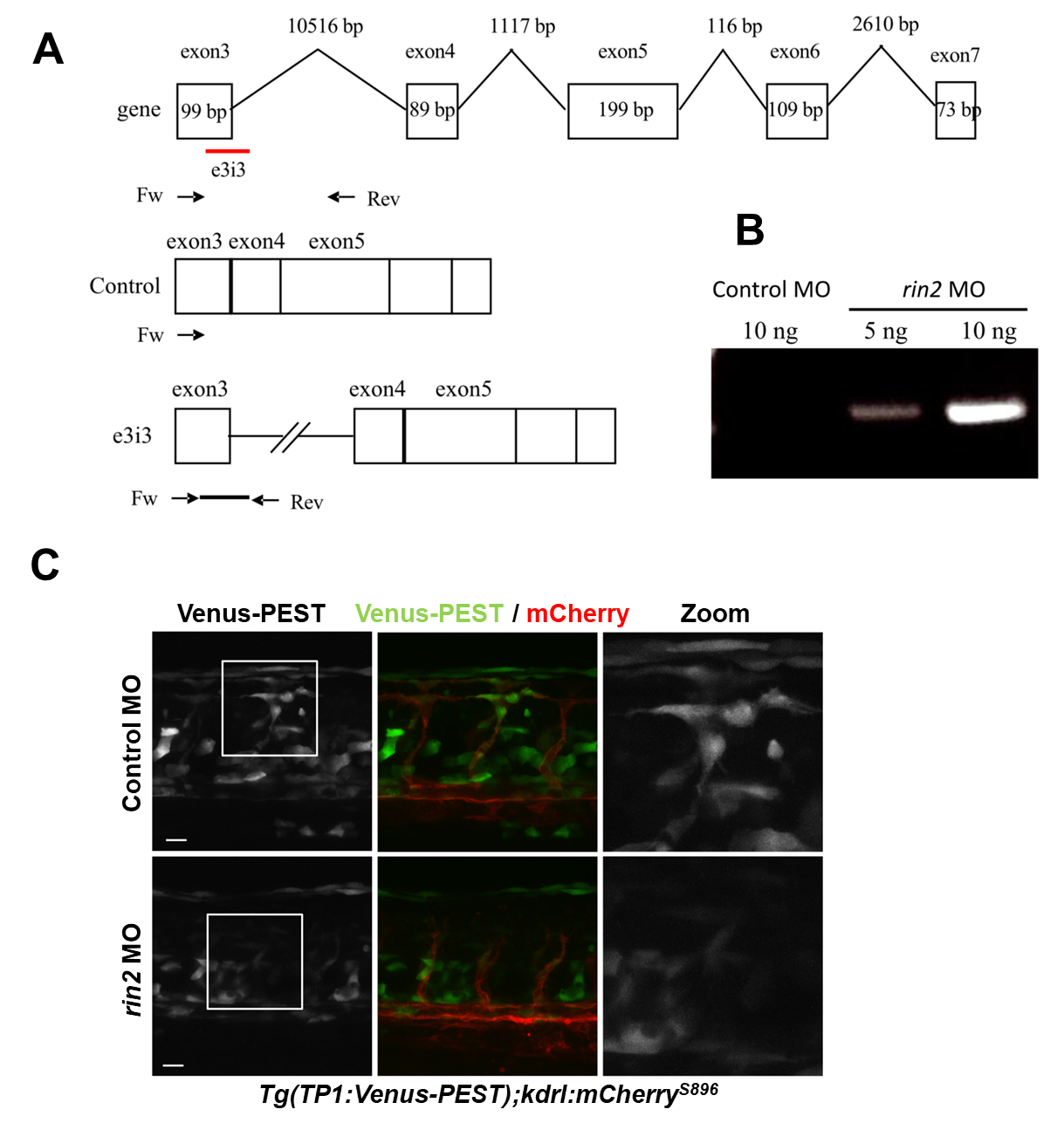

Supplement: Supplementary file 7 — Electronic supplementary material 7 (TIF 707 kb) [file 10456_2021_9788_MOESM7_ESM.tif]
