# Supplementary material for: The endosomal RIN2/Rab5C machinery prevents VEGFR2 degradation to control gene expression and tip cell identity during angiogenesis
Source: Angiogenesis. 2021 May 13;24(3):695–714. doi: 10.1007/s10456-021-09788-4 (PMC8292304; doi:10.1007/s10456-021-09788-4)
Supplement: Supplementary file 8 — Electronic supplementary material 8 (DOCX 34 kb) [file 10456_2021_9788_MOESM8_ESM.docx]

**Supplemental Figure legends**

**Supplemental Figure 1.** **Knockdown efficiency, VEGFR1 expression, and effects of lysosomal inhibitors on VEGFR2 degradation.** (**A**) Relative mRNA expression of Rab5C in HUVECs lentivirally transduced with *RAB5C*-targeting shRNAs was assessed by qPCR and compared to that in HUVECs transduced with a scrambled sequence (set to 100%). Expression is normalized to that of actin, and results are means + SEM of 4 independent experiments. (**B**) Relative VEGFR1 levels quantified from Western blots and normalized to total protein content. Levels in sh_Ctrl cells were set to 1. Results are means + SEM of 3-4 independent experiments. (**C**) HUVECs were serum-starved overnight, pre-treated for 30 mins in serum-free medium with Bafilomycin (100nM), Leupeptin (10µM), and Pepstatin (10µM), and subsequently stimulated with 50 ng/ml VEGF for the indicated time-points in the continued presence of the inhibitors. Lysates were subjected to Western blot analysis for VEGFR2, using α-tubulin as a loading control. Quantification shows the decline in VEGFR2 levels, expressed relative to the levels at t=0. Bars represent means + SEM of 3 independent experiments.

**Supplemental Figure 2. Rab5C promotes tip cell formation.** (**A**) Knockdown efficiency of two individual shRNAs targeting Rab5C in HUVECs was assessed by Western blotting, using α-tubulin as a loading control. (**B**) sh_Ctrl and sh_Rab5C cells were differentially labeled using CellTracker dyes, whereafter they were mixed in a 1:1 ratio on beads and subjected to VEGF-stimulated sprouting in fibrin gels. After 48 hours, cells were fixed and processed for confocal microscopy, and quantification was performed by determining the number of tip cells from confocal z-stacks, normalized to the total number of cells in that color. Results shown are the means + SEM, from 3 pooled independent experiments. Around 10 beads (containing on average 10 tip cells/bead) were analyzed per condition per experiment. (**C**) Representative merged and single-channel images from mosaic sprouting assays to determine tip cell formation by sh_Ctrl (*green*) or sh_Rab5C cells (*pseudocolored blue*), using two different hairpins against Rab5C. Images show maximum projections of z-stacks, with nuclei stained by Hoechst (*white*). Scale bar, 75 μm.

**Supplemental** **Figure 3.** **DN mutations impair Rab5C recruitment to endosomes.** (**A**) Sequences of the GTP-binding site in human WT and DN Rab5C. The boxed residue is essential for GTP binding and has been substituted in the DN mutant as indicated. (**B**) Subcellular localization of mCherry-tagged WT and DN Rab5C (*red*) in HUVECs, co-stained for EEA-1 or TGN46 (*green*) and visualized by confocal microcopy. Nuclei are stained with Hoechst (*blue*). Scale bar, 20 μm.

**Supplemental Figure 4. Rab5C regulates tip cell formation *in vivo*.** Embryos mosaically expressing mCherry, mCherry-WT-Rab5C, or mCherry-DN-Rab5C were analyzed for the position of the mCherry-labeled endothelial cells (*red*) in the ISVs (*green*) at 30-32 hpf. (**A**) Maximum projection of a confocal z-stack of a *Tg(kdrl:GFP)^s843^* embryo at 30 hpf, indicating the vessel location scored for mCherry-positive cell positioning. DA, dorsal aorta; ISV, intersegmental vessel; arrowhead, tip cell; arrow, stalk cell. Scale bar, 20 μm. (**B**) Graph showing the distribution (tip, stalk, or DA) of mCherry-positive endothelial cells of embryos mosaically expressing mCherry, mCherry-WT-Rab5C, or mCherry-DN-Rab5C (mCherry: *N*=12 embryos, *n*=342 cells; mCherry-WT-Rab5C: *N*=12, *n*=419; mCherry-DN-Rab5C: *N*=14, *n*=253). Percentages were calculated per embryo, shown are the mean values. (**C**) Subcellular distribution of mCherry-tagged WT and DN Rab5C in the ISVs of *Tg(fli1a:mCherry-WT-hRAB5C)^mu227^;Tg(kdrl:GFP)^s843^* and *Tg(fli1a:mCherry-DN-hRAB5C)^mu228^;Tg(kdrl:GFP)^s843^* zebrafish embryos, visualized by confocal microcopy. Scale bar, 20 μm. (**D**) Quantification of the number of vesicles positive for mCherry in *Tg(kdrl:GFP)^s843^* zebrafish embryos expressing mCherry, mCherry-WT-Rab5C, or mCherry-DN-Rab5C (*n*=5 cells per condition).

**Supplemental Figure 5. Design of a *rab5c* splicing MO and strategy to generate *rab5c^mu229/+^* zebrafish by CRISPR/Cas9.** (**A**) A *rab5c* splice-blocking MO (*red line*) was designed that targets the boundary between exon2 and intron2. Schematic diagrams indicate the *rab5c* gene, normally-spliced mRNA (Control), and aberrantly-spliced mRNA (e2i2) produced in a *rab5c* MO-injected embryo. (**B**) RT-PCR result confirming the efficiency of the e2i2 splice-blocking MO against *rab5c* using primers indicated in (**A**). (**C**) Western blot confirming the efficiency of the *rab5c* translation MO. (**D**) Sequencing of *rab5c^mu229/+^* reveals a 10 bp deletion, which generates a premature stop codon (**E**), thus terminating protein synthesis at 29 (instead of 221) amino acids.

**Supplemental Figure 6. Time-lapse analysis of sprouting, generation of the CA-Rab5C mutant, and effects of Rab5C knockdown on RIN2 expression in HUVECs.** (**A**) Maximum projections of confocal z-stacks obtained at the indicated time-points by time-lapse analysis in *Tg(kdrl:GFP)^s843^* embryos injected with either Control MO (*top*) or *rab5c* ATG MO (*bottom*). Scale bars, 10 μm. (**B**) Sequences of human WT and CA Rab5C. The boxed residue has been substituted in the CA mutant as indicated. (**C**) Subcellular localization of mScarlet-tagged CA Rab5C (*red*) in HUVECs, co-stained for EEA-1 (*green*) and visualized by confocal microcopy. Nuclei are stained with Hoechst (*blue*). Scale bar, 10 μm. (**D**) RIN2 expression in Rab5C-depleted HUVECs. Representative Western blots (*left*) and quantification of RIN2 levels (*right*). Graphs show averages + SEM from n=3.

**Supplemental Figure 7. Generation of *rin2* splice-blocking MO and activation of Notch signaling in MO-injected embryos.** (**A**) A *rin2* splice-blocking MO (*red line*) was designed that targets the boundary between exon3 and intron3. Schematic diagrams indicate the *rin2* gene, normally-spliced mRNA (Control), and aberrantly-spliced mRNA (e3i3) produced in a *rin2* MO-injected embryo. (**B**) RT-PCR confirming the efficiency of the *rin2* splice-blocking MO using primers indicated in (**A**). (**C**) *Tg(TP1:Venus-PEST);(kdrl-mCherry)^s896^* zebrafish embryos demonstrating activation of Notch signaling in embryos injected with Control MOs (*top*), which is reduced in embryos injected with *rin2* MOs (*bottom*). Scale bars, 20 μm.

**Supplemental tables**

**Supplemental Table 1. The ‘VEGF transcriptome’ as determined by RNA-seq.** HUVECs were deprived of growth factors overnight, then stimulated with VEGF for 1 hour. Shown are the genes (in alphabetical order) whose expression is significantly different at t=1 versus t=0 in sh_Ctrl HUVECs (adjusted *p* value<0.05), based on data from 2 independent experiments. Indicated is the overlap with VEGF transcriptomes in previous studies, and genes encoding transcriptional regulators are highlighted in yellow. FC, fold change.

**Supplemental Table 2. Expression of Rab5 GEFs in HUVECs.** Black and red expression values indicate significant and absent expression, respectively. Column A, Gene name as annotated in NCBI_Gene; Column B, Gene ID as annotated in NCBI_Gene; Column C, alternative gene names as annotated in NBCI_Gene; Column D, Affymetrix probe number; Column E, Probes ranked by product size (descending order); Column F, Average expression value in HUVEC-29 as determined with MAS5.0 analysis; Column G, Significant probe expression with MAS5.0 (n = number of samples with expression, 17 maximum); Column H-AK, Dataset GSE ID, PubMed ID, GSM ID, MAS5.0 yes/no.

**Supplemental Table 3. List of primers used in this study for qPCR.**

**Supplemental Table 4. List of shRNA sequences used in this study.**

**Supplemental Table 1**

| **VEGF-induced genes** **at t=1** | | | | | **Overlap with previous studies** | | |
| --- | --- | --- | --- | --- | --- | --- | --- |
| **hgnc_symbol** | **Ensembl_gene_id** | **Entrez gene** | **LogFC** | **Adjusted p-value** | **Fish et al., 2017** | **Schweighofer et al., 2009** | **Shin et al., 2008** |
| *AHDC1* | ENSG00000126705 | 27245 | -0.491188652 | 0.048641746 |  |  |  |
| *ARL4C* | ENSG00000188042 | 10123 | 0.620160812 | 0.018797292 |  |  |  |
| *ATF3* | ENSG00000162772 | 467 | 2.366105771 | 0.001071673 | X | X | X |
| *BACH1* | ENSG00000156273 | 100379661//571 | 0.527052174 | 0.021812813 |  |  |  |
| *BHLHE40* | ENSG00000134107 | 8553 | 1.090388097 | 0.002301382 | X |  |  |
| *CD83* | ENSG00000112149 | 9308 | 2.381698741 | 0.027531941 | X |  |  |
| *CSRNP1* | ENSG00000144655 | 64651 | 1.768237524 | 0.017436508 | X |  |  |
| *DUSP5* | ENSG00000138166 | 1847 | 0.389890028 | 0.032445896 | X | X | X |
| *EFNB2* | ENSG00000125266 | 1948 | 0.453005009 | 0.028954083 |  |  | X |
| *EGR1* | ENSG00000120738 | 1958 | 4.785152688 | 0.001540241 | X | X | X |
| *EGR2* | ENSG00000122877 | 1959 | 7.245986011 | 0.002982979 | X | X | X |
| *EGR3* | ENSG00000179388 | 1960 | 2.303457047 | 5.63E-05 | X | X | X |
| *EGR4* | ENSG00000135625 | 1961 | 5.065294157 | 0.014872277 | X |  |  |
| *F3* | ENSG00000117525 | 2152 | 3.459126232 | 9.37E-05 | X | X | X |
| *FOS* | ENSG00000170345 | 2353 | 4.337100389 | 0.001957554 | X | X | X |
| *FOSB* | ENSG00000125740 | 2354 | 3.261655566 | 0.001540241 | X | X | X |
| *FOXO1* | ENSG00000150907 | 2308 | 0.399162616 | 0.025801268 |  |  |  |
| *GEM* | ENSG00000164949 | 2669 | 2.354026396 | 0.034843189 | X |  |  |
| *HES1* | ENSG00000114315 | 3280 | 1.384072926 | 0.000913311 | X |  | X |
| *HLX* | ENSG00000136630 | 3142 | 1.681394507 | 0.023376492 | X | X |  |
| *ID1* | ENSG00000125968 | 3397 | 1.298182995 | 0.005063383 |  |  |  |
| *JUNB* | ENSG00000171223 | 3726 | 1.435738335 | 0.000443907 | X | X |  |
| *KDM6B* | ENSG00000132510 | 23135 | 0.790788972 | 0.004976812 | X |  |  |
| *KLF10* | ENSG00000155090 | 7071 | 1.225595999 | 0.001032435 | X | X | X |
| *KLF4* | ENSG00000136826 | 9314 | 0.906815557 | 0.017436508 | X | X |  |
| *MAFB* | ENSG00000204103 | 9935 | 1.001988515 | 0.049050814 |  |  | X |
| *MAFF* | ENSG00000185022 | 23764 | 0.597584271 | 0.035392601 | X | X | X |
| *MCL1* | ENSG00000143384 | 4170 | 1.417245699 | 0.032445896 | X |  |  |
| *MIDN* | ENSG00000167470 | 90007 | 0.754273835 | 0.000372727 | X |  | X |
| *NAB2* | ENSG00000166886 | 4665 | 1.551163841 | 5.01E-05 | X | X |  |
| *NR4A1* | ENSG00000123358 | 3164 | 4.788676433 | 2.49E-07 | X | X | X |
| *NR4A2* | ENSG00000153234 | 4929 | 3.983538221 | 2.66E-05 | X | X | X |
| *NR4A3* | ENSG00000119508 | 8013 | 3.856746459 | 4.88E-05 | X | X | X |
| *PER1* | ENSG00000179094 | 5187 | 0.776155125 | 0.002446158 | X | X |  |
| *PIM1* | ENSG00000137193 | 5292 | 1.045682525 | 0.010529703 |  |  |  |
| *PTGS2* | ENSG00000073756 | 5743 | 2.410792097 | 0.002426057 | X | X | X |
| *RCAN1* | ENSG00000159200 | 1827 | 9.356392511 | 5.63E-05 | X | X | X |
| *RRAD* | ENSG00000166592 | 6236 | 1.829831206 | 0.019947973 | X |  | X |
| *ZC3H12A* | ENSG00000163874 | 80149 | 0.756624834 | 0.036564225 | X |  |  |
| *ZFP36* | ENSG00000128016 | 7538 | 1.641216647 | 0.00996993 |  | X | X |
| *ZNF697* | ENSG00000143067 | 90874 | 0.567313719 | 0.012331312 |  |  |  |

**Supplemental Table 2; see Excel file.**

**Supplemental Table 3**

| Gene | Primer | Sequence (5’ to 3’) |
| --- | --- | --- |
| *ACTB* | Fw | CTT CCT TCC TGG GCA TGG AGT C |
|  | Rev | CTC AGG AGG AGC AAT GAT CTT GAT CTT C |
| *ANGPT2* | Fw | GCA AAA TCA GCA GCA TCA GCC AA |
|  | Rev | GCA TCA AAT CAC CAG CCT CCT GT |
| *APLN* | Fw | CCA GAT GAC AAT CAG ACG GAC AG |
|  | Rev | GGC ACC ATT CCA CCA AAA GAT G |
| *DLL4* | Fw | TGT GAC CAA GAT CTC GAC TAC TGC |
|  | Rev | TAG CCA TCC TCT TGG TCC TTA CAG |
| *NID2* | Fw | GGC ACC AGT GAA CTG ATG TAG ATG |
|  | Rev | AA GTT GCC CTG CTC GTC GTA TTG |
| *NRP2* | Fw | GA GCC CTG TGG TTG GAT GTA TG |
|  | Rev | TCA TCT GGA AAC GTC CGG TCG |
| *PDGFB* | Fw | TCC AGG TGA GGA AGA TCG AGA TTG |
|  | Rev | TTT TGG CTC GCT GCT CCT GG |
| *RAB5C* | Fw | TGC CTG GAT GAC ACA ACA GTC AAG |
|  | Rev | TGC AAT GAC GAT GTT GAG GCT GG |
| *VEGFR2* | Fw | CTC TGT GGG TTT GCC TAG TGT TTC TC |
|  | Rev | CTG TCC CCT GCA AGT AGT TTG AAG |
| *VEGFR3* | Fw | GCC AGG TAT TAT AAC TGG GTG TCC |
|  | Rev | TCT GGT TGT CCA CAG AGC CTT TG |

**Supplemental Table 4**

| Target | Oligo ID TRCN0000 | Full hairpin sequence |
| --- | --- | --- |
| *ALS2* | 047803 | CCGGCGACTAAATAAGCAGCCAGATCTCGAGATCTGGCTGCTTATTTAGTCGTTTTTG |
|  | 047804 | CCGGCCTGGATTGTTGTCACAAGTTCTCGAGAACTTGTGACAACAATCCAGGTTTTTG |
|  | 047806 | CCGGGCAGGATTCCAGTTCTTGTTACTCGAGTAACAAGAACTGGAATCCTGCTTTTTG |
| *ALS2CL* | 078728 | CCGGCGCTGTAAAGGACCTTCCATTCTCGAGAATGGAAGGTCCTTTACAGCGTTTTTG |
|  | 078729 | CCGGCCCGAAGAAGAGTTCTCCTTTCTCGAGAAAGGAGAACTCTTCTTCGGGTTTTTG |
|  | 078730 | CCGGCAGGCCCACATAGAGTACATTCTCGAGAATGTACTCTATGTGGGCCTGTTTTTG |
|  | 078731 | CCGGCTGAGGACAAGTTCGACTGTTCTCGAGAACAGTCGAACTTGTCCTCAGTTTTTG |
|  | 078732 | CCGGGCTGCGTAGGTCTCAGGATTACTCGAGTAATCCTGAGACCTACGCAGCTTTTTG |
| *GAPVD1* | 006005 | CCGGGCCACTTTACATGAGCCAATTCTCGAGAATTGGCTCATGTAAAGTGGCTTTTT |
|  | 006006 | CCGGGCAGTTTCTTTATGGTGCAATCTCGAGATTGCACCATAAAGAAACTGCTTTTT |
|  | 006007 | CCGGCGCAGGATTCAGCTTTCTCTTCTCGAGAAGAGAAAGCTGAATCCTGCGTTTTT |
|  | 006008 | CCGGGCAAGCTACAACACAGGATAACTCGAGTTATCCTGTGTTGTAGCTTGCTTTTT |
|  | 010987 | CCGGCCCTTGTTGTTGGGAGCATTTCTCGAGAAATGCTCCCAACAACAAGGGTTTTT |
| *RAB5C* | 380031 | GTACCGGTATGCAGACGACAACAGTTTGCTCGAGCAAACTGTTGTCGTCTGCATATTTTTTG |
|  | 310771 | CCGGTCATTGCACTCGCGGGTAACACTCGAGTGTTACCCGCGAGTGCAATGATTTTTG |
|  | 380897 | GTACCGGGCTTTGTCAAGGGACAGTTTCCTCGAGGAAACTGTCCCTTGACAAAGCTTTTTTG |
| *RABGEF1* | 047233 | CCGGCGTCAAGCAAATGTATAAGAACTCGAGTTCTTATACATTTGCTTGACGTTTTTG |
|  | 047234 | CCGGCGATCACAGATATCATTGAAACTCGAGTTTCAATGATATCTGTGATCGTTTTTG |
|  | 047235 | CCGGGTCCTTCCATAAACCGGCAAACTCGAGTTTGCCGGTTTATGGAAGGACTTTTTG |
|  | 047236 | CCGGGTTCAAGACATCGTTGAGAAACTCGAGTTTCTCAACGATGTCTTGAACTTTTTG |
|  | 047237 | CCGGCCAGAAAGAGTCGAGAAGATACTCGAGTATCTTCTCGACTCTTTCTGGTTTTTG |
| *RIN1* | 077883 | CCGGGAGCCTGCTGAGAAATCCTTTCTCGAGAAAGGATTTCTCAGCAGGCTCTTTTTG |
|  | 077884 | CCGGCCACTGTATGACGTGCCCAATCTCGAGATTGGGCACGTCATACAGTGGTTTTTG |
|  | 077885 | CCGGCAGTCTGAGACAACTGCTGAACTCGAGTTCAGCAGTTGTCTCAGACTGTTTTTG |
|  | 077886 | CCGGGAGAAGTCATTGCATTGCTCTCTCGAGAGAGCAATGCAATGACTTCTCTTTTTG |
| *RIN2* | 062644 | CCGGCGCATCAAGAACGATCCTTATCTCGAGATAAGGATCGTTCTTGATGCGTTTTTG |
|  | 062645 | CCGGGCAGGGATGTTCTACCATTTACTCGAGTAAATGGTAGAACATCCCTGCTTTTTG |
|  | 062647 | CCGGCCTGGTTCATAAATCTACCAACTCGAGTTGGTAGATTTATGAACCAGGTTTTTG |
| *RIN3* | 077863 | CCGGCGAGACGGCATGTTCTTCATTCTCGAGAATGAAGAACATGCCGTCTCGTTTTTG |
|  | 077864 | CCGGGCTCAAGACCTGCAAACTCATCTCGAGATGAGTTTGCAGGTCTTGAGCTTTTTG |
|  | 077865 | CCGGGCCTGTGGTTTGTGAATCCTACTCGAGTAGGATTCACAAACCACAGGCTTTTTG |
|  | 077866 | CCGGGAGGACATCTTCAGATTGATTCTCGAGAATCAATCTGAAGATGTCCTCTTTTTG |
|  | 077867 | CCGGGCACATCAAGAGCTACGACAACTCGAGTTGTCGTAGCTCTTGATGTGCTTTTTG |
| *RINL* | 161025 | CCGGGCTGGACGTAGAGTTTCTTATCTCGAGATAAGAAACTCTACGTCCAGCTTTTTTG |
|  | 162232 | CCGGCGTAGAGTTTCTTATGGAGCTCTCGAGAGCTCCATAAGAAACTCTACGTTTTTTG |
|  | 163218 | CCGGGAGGTGTGCAGAGATGTCTATCTCGAGATAGACATCTCTGCACACCTCTTTTTTG |
|  | 163219 | CCGGGCTTTCCATTGTGAACCAGCTCTCGAGAGCTGGTTCACAATGGAAAGCTTTTTTG |
|  | 164177 | CCGGCAGCCTGACAAACATGGAGAACTCGAGTTCTCCATGTTTGTCAGGCTGTTTTTTG |
|  | 163681 | CCGGGACCTTGAAGGAAAGGAGGAACTCGAGTTCCTCCTTTCCTTCAAGGTCTTTTTTG |
|  | 159236 | CCGGCAAAGAATGTATCACATGCTACTCGAGTAGCATGTGATACATTCTTTGTTTTTTG |
|  | 161859 | CCGGGAAGGTGCTTTCCATTGTGAACTCGAGTTCACAATGGAAAGCACCTTCTTTTTTG |
|  | 160338 | CCGGCACATGCTAACAAACCATATACTCGAGTATATGGTTTGTTAGCATGTGTTTTTTG |
| Scrambled |  | CCGGCAACAAGATGAAGAGCACCAACTCGAGTTGGTGCTCTTCATCTTGTTGTTTTT |

**Legends for video files**

**Supplemental Movie 1. Normal ISV sprouting in zebrafish.** Confocal time-lapse movie of ISV formation in a representative *Tg(kdrl:GFP)^s843^* embryo injected with Control MO. Movie started at 20 hpf. Confocal images were acquired in 15 min intervals during 6 hrs.

**Supplemental Movie 2. ISV sprouting in zebrafish depleted of Rab5c.** Confocal time-lapse movie of ISV formation in a representative *Tg(kdrl:GFP)^s843^* embryo injected with *rab5C* ATG MO. Movie started at 20 hpf. Confocal images were acquired in 15 min intervals during 6 hrs.

**Supplemental Movie 3. Filopodia formation during ISV sprouting in zebrafish.** Confocal time-lapse movie of filopodia formation in a representative *Tg(fli1a:lifeact-GFP)* embryo injected with Control MO. Movie started at 28 hpf. Confocal images were acquired in 6 min intervals during 1 hr.

**Supplemental Movie 4. Filopodia formation during ISV sprouting in zebrafish depleted of Rab5c.** Confocal time-lapse movie of filopodia formation in a representative *Tg(fli1a:lifeact-GFP)* embryo injected with *rab5c* ATG MO. Movie started at 28 hpf. Confocal images were acquired in 6 min intervals during 1 hr.
